# ﻿The genus *Plagiothecium* (Plagiotheciaceae) in Europe — current state of knowledge, checklist and key to taxa

**DOI:** 10.3897/phytokeys.253.142268

**Published:** 2025-03-04

**Authors:** Grzegorz J. Wolski

**Affiliations:** 1 Department of Geobotany and Plant Ecology, Faculty of Biology and Environmental Protection, University of Lodz, Banacha 12/16, 90-237 Lodz, Poland University of Lodz Lodz Poland

**Keywords:** Distribution, lectotype, new combinations, new synonyms, new taxa, resurrection

## Abstract

This manuscript presents current knowledge of the taxonomy, variability and distribution of taxa of the genus *Plagiothecium* in Europe. Currently the genus comprises 31 taxa: 17 species and 14 varieties. In this study I designated 10 lectotypes and proposes six new synonyms for the described taxa, in addition five new nomenclatural combinations: P.denticulatumvar.pseudosylvaticum, P.denticulatumvar.pungens, P.laetumvar.hercinicum, P.succulentumvar.cryptarum, and P.sylvaticumvar.immersum. Detailed descriptions and distribution data for each taxon, illustrations highlighting key taxonomic features and a diagnostic key are also provided to facilitate the identification of individual taxa.

## ﻿Introduction

*Plagiothecium* Schimp. is a pleurocarpous genus with a global distribution, with the most frequently recorded and most widespread species being in the Northern Hemisphere (e.g., Jedlička 1948, 1950, 1961; Sakurai 1949; Ireland 1969, 1985, 1992; Iwatsuki 1970; Lewinsky 1974; Ireland and Buck 1994; Ochyra et al. 2008; Wynns et al. 2017; Wolski et al. 2020, 2021a, 2022a, b, 2024). In the Southern Hemisphere, there are significantly fewer species, but as recent studies indicate, this is still an area with an under-recorded number of taxa of this genus (Wolski et al. 2024).

This genus was first described in *Bryologia Europea* (Bruch et al. 1851). Since then, due to its usually medium to large size and very characteristic, flattened habit, it has been an included element of all bryological revisions and monographs (e.g., Paris 1894–1898; Dixon 1904; Brotherus 1923; Mönkemeyer 1927; Grout 1932; Podpěra 1954; Szafran 1960) and has never been omitted by bryologists.

Throughout history, the genus *Plagiothecium* has also undergone a relatively large number of national or continental revisions (Jedlička 1948, 1950, 1961; Sakurai 1949; Greene 1957; Ireland 1969, 1985, 1992; Iwatsuki 1970; Lewinsky 1974; Buck and Ireland 1989; Ireland and Buck 1994; Buck 1998; Li and Ireland 2011; Ignatova et al. 2019; Wolski and Nowicka-Krawczyk 2020; Wolski et al. 2022a, b, 2024). An attempt to revise this genus on a global scale was also made (Wynns 2015). However, such a broad approach will not provide an accurate picture of the relations between individual taxa, because, as recent years of research have shown, almost every epithet in this genus, and every complex, requires verification of its taxonomic status (Wolski and Nowicka-Krawczyk 2020; Wolski and Proćków 2020, 2021; Wolski et al. 2021a, 2022a-b, 2024).

Revisions by earlier scientists (Jedlička 1948, 1950, 1960; Sakurai 1949; Greene 1957; Ireland 1969, 1985, 1992; Iwatsuki 1970) had a great influence on later generations of bryologists, shaping in a certain way the perceptions of the genus. Most importantly, the above-mentioned articles also influence our current perception of individual taxa of *Plagiothecium* (e.g., Lewinsky 1974; Noguchi 1994; Smith 2001; Cano 2018; Li and Ireland 2011).

Revisions made in the previous century resulted in the fact that in the history of this genus we can distinguish two periods — the first one connected with the multiplication of the number of individual taxa (e.g., Jedlička 1948, 1950, 1960; Sakurai 1949), and the second one, initiated independently by Ireland (1969, 1985) and Iwatsuki (1970) — the reductionist period. The reduction of the number of taxa was connected with the mass synonymization of individual names in *Plagiothecium*. It led to the fact that in Europe, out of 117 taxa distinguished by Jedlička (1948, 1950), within about 25 years, Lewinsky (1974) reported only 11 species. This idea and reductionist approach was adopted by subsequent researchers and for decades was widely accepted by bryologists (Noguchi 1994; Smith 2001; Iwatsuki 2004; Suzuki 2016; Cano 2018).

This overly broad treatment of individual taxa of *Plagiothecium* resulted in individual researchers very often pointing out that species within the genus are highly variable and cause a number of taxonomic difficulties (Nyholm 1965; Noguchi 1994; Smith 2001; Wolski 2017, 2018; Cano 2018).

The latest literature (Wynns et al. 2017; Wolski and Nowicka-Krawczyk 2020; Wolski et al. 2020, 2021b, 2022a, b, c, 2024) presents a balanced approach, which does not align very closely with the reductionist vision of most predecessors, nor with the synonymizations proposed by them (e.g., Ireland 1969, 1985; Iwatsuki 1970; Lewinsky 1974). Thus, the aforementioned studies not only resurrected a number of previously synonymized taxa, but also allowed for the description of new species. This clearly indicates that the number of taxa from individual continents is greatly under-estimated (Wynns et al. 2017; Wolski and Nowicka-Krawczyk 2020; Wolski et al. 2020, 2021b, 2022a, b, c, 2024).

Taking into account the above and the relatively rapid changes in the taxonomy of *Plagiothecium*, the aim of the following manuscript is to collect all current knowledge on the genus in Europe, to create a checklist of the accepted infrageneric taxa and to present a key for the identification of European taxa.

## ﻿Materials and methods

The following study, including the data contained therein, is part of the results obtained from my ongoing revision of the genus *Plagiothecium* since 2016. The results below are a compilation of my published works (Wolski 2020; Wolski and Nowicka-Krawczyk 2020; Wolski and Proćków 2020, 2021, 2022; Wolski et al. 2020, 2021a, 2022 a-d) as well as my unpublished data.

The conducted research and revision were based on herbarium collections from 52 world herbaria (AAU, B, BG, BM, BRA, BRNU, C, CP, E, F, FH, G, GB, H, HBG, JE, IBL, KRAM B, LBL, LOD, M, MANCH, MICH, MO, MU, NTNU, NY, OXF, PL, POZG-B, PC, PR, PRC, S, SLO, SOSN, SZUB-B, TAA, TALL, TAM, TRH, TROM, TU, TUB, TUR, UBC, UME, UPS, YU, VLA, WRSL), including the study of 90 nomenclature types of this genus.

The division of species according to cell areolation was made according to the width of the cells from the middle part of the leaf. Whereby when the cells were 7–9 µm wide, areolation was recognized as tight; cells 11–15 µm wide are termed quite loose; cells 16–19 µm are referred to as loose; while with cells above 20 µm wide, areolation was considered as very loose.

Data on the geographical distribution of individual taxa were taken from the labels of herbarium specimens and were supported by literature data.

## ﻿Results

Currently, in Europe, within the genus *Plagiothecium*, 31 taxa can be distinguished, belonging to eight sections. The most speciose are the sections *Orthophyllum* Jedl. (11 taxa) and *Leptophyllum* Jedl. (nine). On the other hand, the least speciose are four sections: *Philoscia* (Berk.) Ochyra, *Rectithecium* (Hedenäs and Huttunen) J.T.Wynns, *Pseudo-Neckera* (Kindb.) J.T.Wynns and *Lycambium* Jedl. (each with a single species).

The results of this research not only allows for the proposal of five new combinations, the designation of 10 lectotypes and the proposal of six new synonyms, but also shows that the diversity of *Plagiothecium* in Europe is still under-estimated.

### ﻿Detailed description of individual taxa

#### Sect. Plagiothecium

##### ﻿ Plagiothecium denticulatum var. denticulatum

Taxon classificationPlantaeHypnalesPlagiotheciaceae

(Hedw.) Schimp., Bryologia Europea 5: 190, 501, Tab. VIII. 1851.

B3C71BC5-B7D8-5C56-AECA-EC85522B6184

≡ Hypnumdenticulatum Hedw., Species Muscorum Frondsorum 237. 1801 ≡ Stereodondenticulatus (Hedw.) Brid., Bryologia Universa 2: 824. 1827 ≡ Pancoviadenticulata (Hedw.) J.Kickx f., Flore Cryptogamique des Flandres 1: 93. 1867. Lectotype (designated by Ireland 1969): Germany, *Starke*, G 000420240!
= Plagiotheciumdenticulatumvar.bullulae Grout, North American Musci Perfecti 450 1942. Lectotype (designated here): U.S.A., Idaho, Elmore Co., Boise National Forest, on soil and base of saplings by small water course above cemetery, 22 Sep. 1942, *F. A. MacFadden*, C-M-9386! Isolectotypes: MO-406576, NY 505676, NY 507145.
= Plagiotheciumsylvaticumvar.rupestre Warnst. *ex* Grav., Bulletin de la Société Royale de Botanique de Belgique 19: 31. 1880. Lectotype (designated here): Germany, Bavar. Australis, ad rupes silic. umbros. montium editiorum Silvae Gabretae, parietes verticals investiens, ca. 800–1000 m, Aug 1879, sub “P.silvaticumvar.rupestre*Progel*”, *Progel*, PC 0132568! Isolectotypes: Germany Baiern, Waldmünchen am Böhmerwald, auf Gneissfelsen im Juni, *Progel*, PC 0132569! syn. nov.

###### Description.

**Figure 1.**
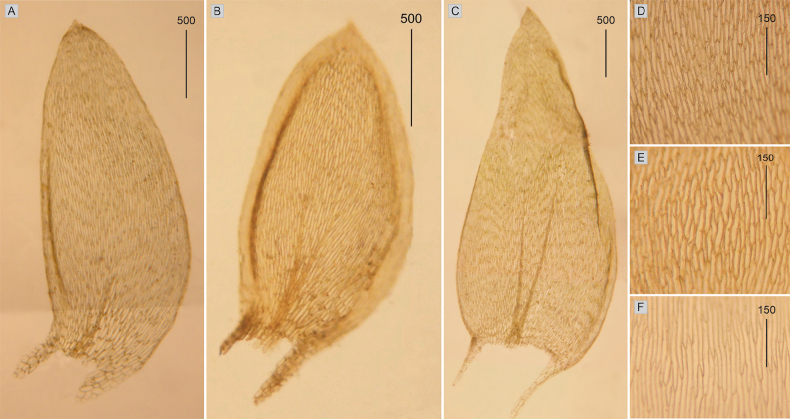
Selected, most important taxonomic features of taxa from the *Plagiotheciumdenticulatum* complex **A–C** shape and dimensions of the leaves **D–F** shape and dimensions of cells from the middle part of the leaves **A**, **D**P.denticulatumvar.denticulatum (from type of P.denticulatumvar.bullulae, *F. A. MacFadden*, C-M-9386!) **B**, **E**P.denticulatumvar.obtusifolium (from epitype of P.denticulatumvar.obtusifolium, *D. Templeton*, BM 000890810!) **C**, **F**P.denticulatumvar.undulatum (from samples of *P.ruthei*, *S. Lisowski*, POZN-B 12388!).

Plants medium-sized, light to dark green, with metallic luster; stems 2–5 cm long; leaves complanate, more julaceous in lower part of stem, concave, ovate, asymmetrical, with two rounded sides, rounded asymmetric, 1.5–3.0 × 0.5–2.0 mm (Fig. 1A); the apex acute to acuminate; margins denticulate near the apex; laminal cells 80–130 × 10–14 μm at midleaf (Fig. 1D), cell areolation quite loose; decurrencies well developed, consisting of 4–5 rows of spherical, inflated cells; capsule inclined.

###### Distribution.

Asia (Azerbeijan, Bangladesh, China, Democratic People’s Republic of Korea, Iraq, India, Islamic Republic of Iran, Japan, Kazakhstan, Kyrgystan, Mongolia, Nepal, Pakistan, Republic of Korea, Russian Federation); Europe (Albania, Andorra, Armenia, Austria, Belarus, Belgium, Bosnia and Herzegovina, Bulgaria, Croatia, Czech Republic, Denmark, Estonia, Finland, France, Germany, Greece, Hungary, Iceland, Ireland, Italy, Kosovo, Latvia, Lichtenstein, Lithuania, Luxembourg, Montenegro, Netherlands, North Macedonia, Norway, Poland, Portugal, Romania); North America (Canada, U.S.A.).

##### ﻿ Plagiothecium denticulatum var. obtusifolium

Taxon classificationPlantaeHypnalesPlagiotheciaceae

(Turner) Moore, Proceedings of the Royal Irish Academy 1: 424. 1873.

E4DA3943-3500-541D-B0B0-4A43382C5EAB

**Figure 2.**
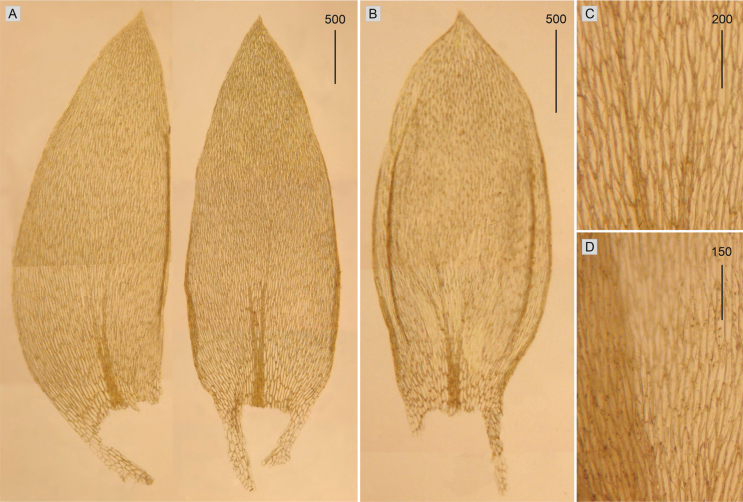
Selected, most important taxonomic features of taxa from the *Plagiotheciumdenticulatum* complex **A, B** shape and dimensions of the leaves **C, D** shape and dimensions of cells from the middle part of the leaves **A**, **C**P.denticulatumvar.pseudosylvaticum (from lectotype of *P.pseudosylvaticum*, *C. Warnstorf*, C-M-9394!) **B**, **D**P.denticulatumvar.pungens (from lectotype of P.silvaticumfo.pungens, *W. Mönkemeyer*, C-M-9396!).

≡ Hypnumdenticulatumvar.obtusifolium Turner, Muscologiae Hibernicae Spicilegium 146, T. 12, f. 2. 1804 ≡ Hypnumobtusifolium (Turner) Brid., Muscologiae Recentiorum Supplementum 2: 93. 1812 ≡ Stereodondenticulatusvar.obtusifolius (Turner) Brid., Bryologia Universa 2: 824. 1827 ≡ Plagiotheciumobtusifolium (Turner) J.J.Amann, Mémoire de la Société Vaudoise des Sciences Naturelles 3: 61. 1928. Holotype: figure 2, tabela 12 “T. 12, f. 2”, Turner 1804: 237. Epitype (designated by Wolski et al. 2022d): [Ireland,] in summo montis Bulbein jugo, ab oculatissimo *D. Brown* lectam, benigne communicavit *D. Templeton*, BM 000890810!
= Plagiotheciumsandbergii Renauld & Cardot, Contributions from the United States National Herbarium 3: 274. 1895. Lectotype (designated by Wolski et al. 2022d): U.S.A., Idaho, Kootenai County, Hope, J. H. *Sandberg*, *D. T. Macdougal*, *A. A. Heller 1174*, August 1892, PC 0132604! Isolectotypes: NY 507114! (available online), US 70396! (available online), FH 220148. Additional original material from *locus classicus* (not signed “*No. 1174*”), NY 507115! (available online); additional *Sandberg* material, potentially from *locus classicus* PC 0132605! and *Sandberg* material FH 220147.
= Plagiotheciumdenticulatumvar.auritum Kern, Jahresbericht der Schlesischen Gesellschaft für Vaterländische Cultur 91(Abt. 2b): 97. 1914. Lectotype (designated by Wolski et al. 2022d): [Italy,] South Tirol, Ortler, Martelltal, in Felshöhlungen oberhalb der Cevedalehütte, 2350 m, 30 July 1913, *F. Kern s.n*., herb. *I. Thériot*, PC 0132639!

###### Description.

Plants small, light green, with metallic luster; stem 0.9–2.5 cm; leaves julaceous, very concave, ovate-elliptical, gently asymmetrical, 1.0–2.2 × 0.5–1.2 mm (Fig. 1B); the apex obtuse, not denticulate; laminal cells linear, 50–140 × 10–21 μm at midleaf (Fig. 1E), cell areolation quite loose; decurrencies broad, alar cells rounded.

###### Distribution.

Asia (China, Islamic Republic of Iran, Japan, Nepal, Russian Federation, Turkey); Europe (Austria, Bulgaria, Czech Republic, Finland, France, Germany, Hungary, Iceland, Ireland, Italy, Kosovo, Luxembourg, Montenegro, Netherlands, Poland, Slovenia, Spain, Sweden, Switzerland, Ukraine, United Kingdom); North America (Canada, U.S.A.).

##### ﻿ Plagiothecium denticulatum var. undulatum

Taxon classificationPlantaeHypnalesPlagiotheciaceae

R.Ruthe ex Geh., Revue Bryologique 4: 42. 1877.

99BC7127-3702-5DD4-8796-9AF2CB323FBE

≡ Plagiotheciumruthei Limpr., Die Laubmosse Deutschland, Oesterreichs und der Schweiz 3: 217. 1897 ≡ Plagiotheciumdenticulatumvar.majusfo.undulatm (R.Ruthe *ex* Geh.) C.E.O.Jensen, Skandinaviens Bladmossflora 494. 1939 ≡ Plagiotheciumrutheisubsp.eu-rutheiGiacomini, Istituto Botanico della R. Università R. Laboratorio Crittogamico Pavia, Atti 4: 278. 1947, nom. inval. Type: près de Barwalde, dans la Nouvelle-Marche, *R. Ruthe*, 1873.

###### Description.

Plants medium-sized, light green, glossy; leaves complanate, transversely undulate, ovate to ovate-lanceolate, asymmetric, with one rounded and one flattened side, shrunken when dry, 2.0–2.5 × 1.0–1.2 mm (Fig. 1C); the apex acute to acuminate; margins denticulate near the apex or not; laminal cells 100–160 × 10–17 μm at the midleaf (Fig. 1F), cell areolation quite loose; decurrencies very long, consisting of 2–3 rows of rounded to rounded-rectangular and inflated cells; capsule inclined.

###### Distribution.

Asia (China, Japan, Russian Federation); Europe (Austria, Belarus, Bulgaria, Czech Republic, Denmark, Estonia, Finland, France, Germany, Greece, Hungary, Ireland, Italy, Latvia, Lichtenstein, Lithuania, Luxembourg, Montenegro, Netherlands, Poland, Romania, Slovakia, Spain, Sweden, Switzerland, Ukraine, United Kingdom); North America (Canada).

##### ﻿ Plagiothecium denticulatum var. pseudosylvaticum

Taxon classificationPlantaeHypnalesPlagiotheciaceae

(Warnst. in Schiffner) G.J.Wolski comb. nov.

B3D65982-207C-52AC-B9E0-37757FB17C40

≡ Plagiotheciumpseudosylvaticum Warnst. *in* Schiffner, Österreische Botanische Zeitschrift 48: 428. 1898. Lectotype (designated by Wynns 2015): [Germany,] Brandenburg, Neuruppin, an dem Waldwege zwischen Rottstiel und dem “Stern” auf von einer schwachen Humusdecke überlagertem Sandboden, 24 July 1897, *C. Warnstorf*, C-M-9394! Isolectotype: PC 0132600! Syntypes: [Poland,] in einer etwas schwächeren, bei Swinemünde, *R. Ruthe*; [Germany,] bei Schönebeck a.d. Elbe, Aug. 1892, Fromm. Apparent topotypes: [Germany,] Neuruppin, Osterwald auf Sand, zwischen Rottstiel und dem Stern, 22 July 1900, *C. Warnstorf*, S-B 160601; Sand., auf Waldboden, bei Rottstiel, July 1898, *C. Warnstorf*, PC 0132601!

###### Description.

Plants medium-sized, light green to yellow green, glossy; stems 2.0–2.5 cm; two types of leaves: symmetrical and asymmetrical, the symmetrical ones: ovate-lanceolate, concave, with two rounded sides, rounded symmetric, asymmetrical ones: ovate-lanceolate, concave, with one rounded and one flattened side, both types of leaves identical in size, 2.0–2.5 × 0.8–1.3 mm (Fig. 2A); the apex acuminate, denticulate; laminal cells 100–130 × 15–20 μm at midleaf (Fig. 2C), cell areolation loose; decurrencies long, consisting of 3–5 rows of rounded and inflated cells; capsule inclined.

###### Distribution.

Europe (Czech Republic, Germany, Poland), but the range of this taxon still requires research.

##### ﻿ Plagiothecium denticulatum var. pungens

Taxon classificationPlantaeHypnalesPlagiotheciaceae

(Mönk.) G.J.Wolski comb. nov.

433CF07B-5A97-54CE-904D-262B77E626C7

≡ Plagiotheciumsylvaticumfo.pungens Mönk., Die Laubmoose Europas 865. 1927 ≡ Plagiotheciumdenticulatumfo.pungens (Mönk.) C.E.O.Jensen, Skandinaviens Bladmossflora 494. 1939. Lectotype (designated here): [Denmark,] Bornholm, an feuchten im Echotale bei Almindingen, sub *Plagiothecium Roeseanum* var. *orthocladon* fo. *pungens*, July 1910, *W. Mönkemeyer*, C-M-9396! Isolectotypes: [Denmark,] Bornholm, an feuchten im Echotale bei Almindingen, sub. Plagiotheciumsilvatiucmfo.pungens, July 1910, *W. Mönkemeyer*, HBG-021135!

###### Description.

Plants medium-sized, yellow green to dark green; stems 1.0–2.0 cm, julaceous-foliate; leaves imbricate, concave, ovate, symmetrical, with two rounded sides, rounded symmetric, 2.0–2.5 × 1.0–1.2 mm (Fig. 2C); the apex acute to acuminate; margins denticulate near the apex; laminal cells 110–160 × 15–20 μm at midleaf (Fig. 2D), cell areolation loose; decurrencies well developed, consisting of 4–5 rows of spherical, inflated cells; capsules unknown for now.

###### Distribution.

Europe (Denmark), but the range of this taxon still requires research.

#### Sect. Rostriphyllum Jedl.

##### ﻿ Plagiothecium sylvaticum var. sylvaticum

Taxon classificationPlantaeHypnalesPlagiotheciaceae

(Brid.) Schimp., Bryologia Europea 5: 192, 503. 1851.

EEEE552C-0022-5A98-944E-A1D92C649926

≡ Hypnumsylvaticum Brid., Muscologiae Recentiorum 2(2): 53, 1 f. 5. 1801 ≡ Hypnumdenticulatumvar.sylvaticum (Brid.) Turner, Muscologiae Hibernicae Spicilegium 146. 1804 ≡ Stereodonsylvaticus (Brid.) Brid., Bryologia Universa 2: 825, 1827 ≡ Hypnumdenticulatumsubsp.sylvaticum (Brid.) Boulay, Muscinées de la France, Mousses 85. 1884 ≡ Plagiotheciumdenticulatumsubsp.sylvaticum (Brid.) Dixon, Student’s Handbook of British Mosses 437. 1896. Lectotype (the clump at the top of the sheet, selected by Iwatsuki 1970): [Germany,] saltus Thuringicus in paluda, *ex* herb. Brid., B 31091501!
= Plagiotheciumsylvaticumvar.flavescens Warnst., Allegmeine Botanische Zeitschrift für Systematik, Floristik, Pflanzengeographie 5(1): 34. 1899. Lectotype (designated here): [Germany,] am Gaisriegl Dreitannenriegel, Bayr Wald, in Quellsümpfen, 1887, *M. Lickleder*, PC 0132583! syn. nov.
= Plagiotheciumplatyphyllum Mönk., Die Laubmoose Europas 866, 207b. 1927 ≡ Plagiotheciumsylvaticumvar.platyphyllum (Mönk.) F.Koppe, Abhandlungen und Berichte der Naturwissenschaftlichen Abteilung der Grenzmärkischen Gesellschaft zur Erforschung und Pflege der Heimat, Schneidemühl 1931 ≡ P.neglectumsubsp.platyphyllum (Mönk.) Szafran, Flora Polska Mchy (Musci) 2: 288, 1961, *comb*. *inval*. Type: Germany, bei Gersfeld in der Rohn 1906, ferner mir aus Thüringen unddem sächsischen Vogtlande unter anderer Bezeichnung bekannt geworden; The Czech Republic, ferner 1911 im Böhmerwalde bei Eisenstein gesammelt. Lectotype (designated by Wolski et al. 2024): Germany, Thüringien, Finsteres Loch, 26 June 1916, *R. Schmidt*, HBG!
= Plagiotheciumrutheifo.submersum Bizot, in sched. Basis: France, Vosges, Hohneck, immergé dans le lac du Frankenthal, *M. Bizot 2910*, PC 0132598!
= Plagiotheciumrutheivar.rivulare Mayl. in sched. Basis: Switzerland, Uri, entre Göschenen et Andermatt, Sep., 1903, *Thériot*, *J. J. Amann*, PC 0132602! syn. nov.

###### Description.

**Figure 3.**
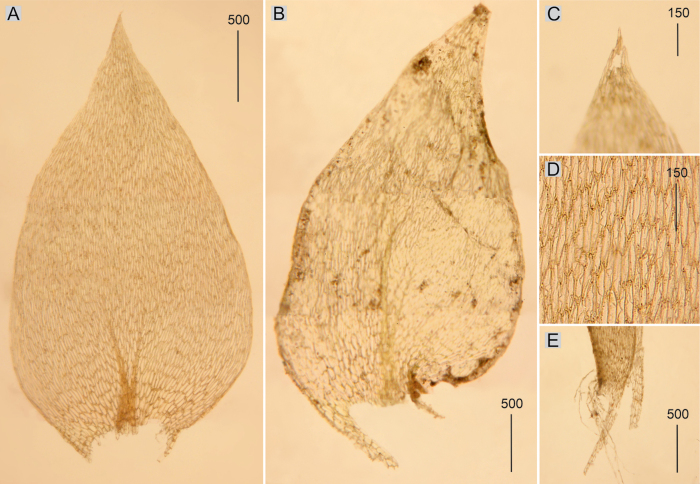
Selected, most important taxonomic features of taxa from the *Plagiotheciumsylvaticum* complex **A, B** shape and dimensions of the leaves **C** eroded leaves apex **D** apex and dimensions of cells from the middle part of the leaves **E** rhizoids on the dorsal side of the leaf **A**, **D**P.sylvaticumvar.sylvaticum (from lectotype of *H.sylvaticum*, *S. É*. *Bridel*, B 31091501!, based on Wolski et al. 2024, changed) **B**, **C**, **E**P.sylvaticumvar.immersum (from lectotype of P.platyphyllumfo.immersa, *Lorrens*, HBG-021127!).

Plants medium-sized to large, light green, dull, without metallic luster; leaves complanate, more or less flat, ovate, not imbricate and not julaceous, symmetrical, 2.0–3.0 × 1.0–1.6 mm (Fig. 3A); the apex acute and denticulate, often eroded; laminal cells 75–160 × 12.5–20 μm at midleaf (Fig. 3D), cell areolation loose; decurrencies long, consisting of 3–4 rows of rounded and inflated cells; capsule inclined.

###### Distribution.

Asia (China, Democratic People’s Republic of Korea, Georgia, Islamic Republic of Iran, Japan, Republic of Korea, Russian Federation, Turkey); Europe (Andorra, Austria, Bulgaria, Croatia, Czech Republic, Denmark, Finland, France, Germany, Greece, Hungary, Ireland, Italy, Kosovo, Lithuania, Luxembourg, Montenegro, North Macedonia, Norway, Poland, Portugal, Romania, Serbia, Slovakia, Slovenia, Spain, Sweden, Switzerland, Ukraine, United Kingdom); North America (Canada, U.S.A.).

##### ﻿ Plagiothecium sylvaticum var. immersum

Taxon classificationPlantaeHypnalesPlagiotheciaceae

(Mönk.) G.J.Wolski comb. nov.

84EF5811-4201-58CF-9821-8E51A5DD990D

≡ Plagiotheciumplatyphyllumfo.immersa Mönk., Die Laubmoose Europas 867. 1927. Type: [Germany,] Aus dem Harze, Thüringen, der Rhön, dem Fichtelgebirge, aus Böhmen, dem Bayerischen Walde, Mähren, der Schweiz (Kanton Uri), Norditalien (Provinz Como) und Bulgarien mir bekannt geworden. Lectotype (designated here): [Switzerland,] Kanton Uri, Schöllenen, 1100–1400 m, 18 August 1884, Lorrens, HBG-021127!

###### Description.

Plants large, dark green, dull, without metallic luster; leaves asymmetrical, complanate, ovate, not imbricate and not julaceous, 3.4–3.6 × 1.4–2.0 mm (Fig. 3B), often with rhizoids on the dorsal side of the leaf (Fig. 3E); the apex acute and denticulate, often eroded (Fig. 3C); laminal cells 90–150 × 8–16 μm at midleaf, cell areolation loose; decurrencies long, consisting of 3–5 rows of rounded and inflated cells; capsule unknown so far.

###### Distribution.

Europe (Bulgaria, Czech Republic, Italy, Switzerland), but the range of this taxon still requires research.

#### Sect. Orthophyllum Jedl.

##### ﻿ Plagiothecium nemorale

Taxon classificationPlantaeHypnalesPlagiotheciaceae

(Mitt.) A.Jaeger, Bericht über die Thätigkeit der St. Gallischen Naturwissenschaftlichen Gesellschaft 1876–1877: 451. 1878.

702C9DAC-7188-5A5A-96AA-EA6D398678F7

≡ Stereodonnemoralis Mitt., Journal of the Proceedings of the Linnean Society, Botany, Supplement 1(2): 104. 1859 ≡ Plagiotheciumsilvaticumvar.nemorale (Mitt.) Paris, Index Bryologicus 967. 1898. Type: Hab. in Himalayae orient. reg. temp., Sikkim, in monte Tonglo (ad radicem filicis cujusdam), *J. D. Hooker*. Lectotype (designated by Wolski et al. 2020): Herb. ind or *Hook*. Fil. & Thomson Stereodonnemorale m. Hab. Sikkim, Tonglo Regio temp. Alt. – *J.D.H*., BM 1030713! Isolectotype: NY 913349!
= Plagiotheciumneglectum Mönk., Die Laubmoose Europas 866. 1927. Lectotype (designated by Wolski and Proćków 2022): figure 207c excluding a part of the figure with the top of the leaf (Mönkemeyer 1927: 862). Epitype (designated by Wolski and Proćków 2022): [Germany,] Wesergebirge, in Erlenbrüchen bei Eschershausen, Juli 1900, *W. Mönkemeyer s.n.* B 300105646! The remaining original material according to Walter and Martienssen (1976) was confirmed to have been lost at HBG: Thüringen: Eisenach, Annatal, 26.7.1898, u. Wartburg, 2.5.1915 (*J. Bornmüller s.n.*); Wesergebirge: Bodenwerder, Königszinne, Juli 1901 (*W. Mönkemeyer s.n.*); Hessen, Rhön: Gr. Nallen, Juli 1906 (*W. Mönkemeyer s.n.*); Vogtland: Plauen, Triebtal, 25.07.1904 (*E. Stolle* s.n.); Bayern: Allgäu, Hinterstein, Sauwald, Aug. 1906, u. Regensburg, U-Lichtenwald, Schindelmacherhänge, Nov. 1906 (*I. Familler s.n.*); Prien/Chiemsee: 500 m, Juni 1911 (*T. Linder s.n.*); Mähren: Oppafall, Juli 1904 (*J. Podpěra s.n.*); Ostpreuβen: Labiau, Juli 1864 (*H. v. Klinggräff s.n.*); Kurland: Usmaitensee, Moritzholm, Mengwald, 3.8.1913 (*K. R. Kupffer s.n.*); sine loc. et dat. (*Wüstnei 380*).
= Plagiotheciumsaxicola Sakurai, Botanical Magazine, Tokyo 48: 395. 1934. Type: [Japan,] Honshu, Prov. Aki, 4 Jan 1933, *Y. Doi 3282*, PC 0132573!

###### Description.

**Figure 4.**
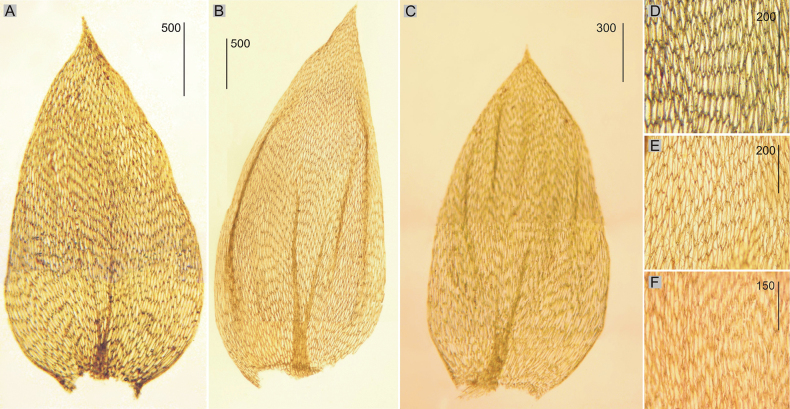
Selected, most important taxonomic features of taxa from the *Plagiotheciumnemorale* complex **A–C** shape and dimensions of the leaves **D–F** shape and dimensions of cells from the middle part of the leaves **A**, **D***P.nemorale* (from lectotype of *S.nemoralis*, *J. D. Hooker*, BM 1030713!, based on Wolski et al. 2020 changed) **B**, **E***P.longisetum* (from lectotype of *P.longisetum*, *S. O. Lindberg*, H-SOL 1563011!) **C**, **F***P.angusticellum* (*B. Goffinet*, *11,795*, NY 02331429, based on Wolski 2020, changed).

Plants medium-sized, dark green, dull, without metallic luster; stems to 1.5–3.0 cm long; leaves complanate, in dry condition shrunken, concave, symmetrical, ovate, those from the middle of the stem 2.2–2.4 × 1.0–1.5 mm (Fig. 4A); the apex acuminate, apiculate and denticulate; laminal cells hexagonal in transverse rows, 50–90 × 17–20 μm at mid-leaf (Fig. 4D), cell areolation loose; decurrencies of 3 rows of rectangular cells; capsule inclined.

###### Distribution.

Asia (Azerbeijan, Bhutan, China, Democratic People’s Republic of Korea, Georgia, India, Islamic Republic of Iran, Japan, Myanmar, Nepal, Pakistan, Philippines, Republic of Korea, Russian Federation, Taiwan, Turkey, Vietnam); Europe (Albania, Andorra, Armenia, Austria, Belarus, Belgium, Bosnia and Herzegovina, Bulgaria, Croatia, Czech Republic, Denmark, Estonia, Finland, France, Germany, Greece, Hungary, Italy, Ireland, Kosovo, Latvia, Lichtenstein, Lithuania, Luxembourg, Montenegro, Netherlands, North Macedonia, Norway, Poland, Portugal, Romania, Serbia, Slovakia, Slovenia, Spain, Sweden, Switzerland, Ukraine, United Kingdom); North America (Canada, U.S.A.).

##### ﻿ Plagiothecium longisetum

Taxon classificationPlantaeHypnalesPlagiotheciaceae

Lindb., Contributio ad Floram Cryptogamam Asiae Boreali-Orientalis 232. 1872 [1873].

56C9DA34-94D2-5809-8A0C-9DFD16BE1C15

= Plagiotheciumroeseanumvar.heterophyllum Warnst., Kryptogamenflora der Mark Brandenburg, Laubmoose 814. 1906 ≡ Plagiotheciumroeseanumfo.heterophyllum (Warnst.) Jedl., Spisy Vydávané Přírodovĕdeckou Fakultou Masarykovy University 308: 40. 1948. Type: Germany, Brandenburgia, Neurippen, Ruppin, auf Waldboden, Böschungen im “Flössergrunde”, *C. Warnstorf*; Westprignitz, Forsthaus “Alte Eiche”, auf Waldboden am Standort von *Osmungaregalis*, Janzen und *C. Warnstorf*; Wittenberge, Westprignitz, am Grunde eines Baumstammes, “Krauses Brack”, *C. Warnstorf*; Ratzburg, Buchenwälder, Prahl. Poland, Świnoujście, Weg nach Corswant, *R. Ruthe* (*n.v*.).
= Plagiotheciummauiense Broth., Bernice P. Bishop Museum Bulletin 40: 28. 1927. Lectotype (designated by Wolski and Proćków 2021): [United States,] Hawaii, E. Maui, Haleakala, 8000 ft., in damp ravines, fertile, June 1876, *D. D. Baldwin 221*, NY 01256708! Isolectotype: FH 00220142!, MU 000000546!, YU 233890!
= Plagiotheciumsylvaticumvar.neglectumfo.orthocladum Barkman, nom. inval., Buxbaumia, 11: 23. 1957. Type: no type was specified.

###### Type.

[Japan,] ad Nikosan ins. Kiusiu, [fertile], 16 Junii 1863, *S. O. Lindberg*. Lectotype (designated by Wolski and Proćków 2020): H-SOL 1563011! Isolectotype: S-B 160017, PC 0132572!

###### Description.

Plants medium-sized to large, green to yellowish, without metallic luster; stems 2–3 cm long; leaves complanate, concave, strongly asymmetrical, ovate to lanceolate, 3.0–4.0 × 1.6–2.0 mm (Fig. 4B); the apex acute to acuminate, not denticulate; laminal cells elongate-hexagonal, in irregular transverse rows, 94–150 × 17–34 μm at midleaf (Fig. 4E), cell areolation very loose; decurrencies of 3 rows of rectangular cells; capsule inclined.

###### Distribution.

Asia (China, Georgia, India, Islamic Republic of Iran, Japan, Nepal, Russian Federation, Turkey); Europe (Austria, Belgium, Denmark, Estonia, Finland, France, Germany, Norway, Poland, Spain, Sweden, Switzerland, United Kingdom); North America (Canada, U.S.A.).

##### ﻿ Plagiothecium angusticellum

Taxon classificationPlantaeHypnalesPlagiotheciaceae

G.J.Wolski & P.Nowicka-Krawczyk, PLoS ONE 15(3): e0230237. 2020.

499C2E5F-F410-542D-B441-CF61F2366790

###### Holotype.

Poland, łódzkie Voivodeship, Grądy nad Moszczenicą reserve, 51°55'N, 19°29'E, at the base of *Carpinusbetulus* in *Fraxino-Alnetum* forest, 11 Dec. 2017, *G. J. Wolski*, LOD 14927! Isotype: LOD 14937!

###### Description.

Plants medium-sized, light to dark green, dull, without metallic luster; stems 2–4 cm long; leaves julaceous and imbricate mainly on lower part of the stem, concave, folded, asymmetrical, ovate to lanceolate, 3.1–3.4 × 1.3–1.5 mm (Fig. 4C); the apex acuminate, short, often gently curved; margins not denticulate near the apex; laminal cells narrowly elongate-hexagonal, 113–143 × 15–19 μm at midleaf (Fig. 4F), cell areolation loose; decurrencies of 3 rows of rectangular to quadrate cells; capsule inclined.

###### Distribution.

Europe (Czech Republic, Estonia, Hungary, Latvia, Lithuania, Poland); North America (U.S.A.).

##### ﻿ Plagiothecium succulentum var. succulentum

Taxon classificationPlantaeHypnalesPlagiotheciaceae

(Wilson) Lindb., Botaniska Notiser 43: 143. 1865.

C134DF7C-252C-5710-AA23-8E67A8A3979E

≡ Hypnumdenticulatumvar.succulentum Wilson, Bryologia Britannica 407. 1855 ≡ Hypnumsucculentum Wilson, Bryologia Britannica 407. 1855, nom. inval. ≡ Plagiotheciumsylvaticumvar.succulentum (Wilson) Spruce, Journal of Botany, British and Foreign 18: 357. 1880 ≡ Plagiotheciumdenticulatumvar.succulentum (Wilson) Dixon, The Student’s Handbook of British Mosses 437. 1896 ≡ Plagiotheciumsylvaticumssp.succulentum (Wilson) Amann & Meyl., Flore des Mousses de la Suisse 1: 174. 1919 ≡ Plagiotheciumlaetumsubsp.succulentum (Wilson) Szafran, Flora Polska Mchy (Musci) 2: 281. 1961. Type: [Great Britain,] Winwick Stone Quarry, near Warrington, *Wilson*; near Todmorden, *J. Nowell*.
= Plagiotheciumsucculentumfo.flavescens Mönk. in sched. Basis: [Denmark,] Insel Bornkolm, bei Helligdommen, Juli 1910, *W. Mönkemeyer*; [Germany,] Fichtelgebirge, unten Bischofsgrün, Juli 1903, *W. Mönkemeyer*; Leipzig, Eilenburg bei Gantsch. Oct. 1905, *W. Mönkemeyer*, HBG! syn. nov.

###### Description.

**Figure 5.**
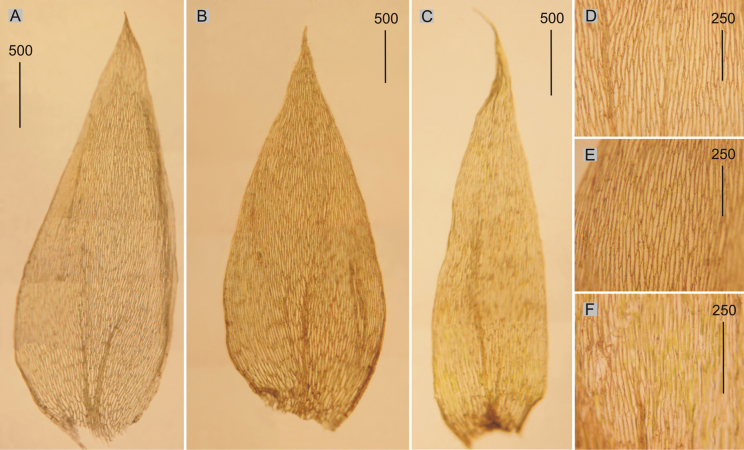
Selected, most important taxonomic features of taxa from the *Plagiotheciumsucculentum* complex **A–C** shape and dimensions of the leaves **D–F** shape and dimensions of cells from the middle part of the leaves **A**, **D**P.succulentumvar.succulentum (*H. N. Dixon*, *B. M. Sutton*, BM 001007959!) **B**, **E**P.succulentumvar.propaguliferum (from lectotype of P.succulentumfo.propaguliferum, *F. V. Schiffner*, C-M-9395!) **C**, **F**P.succulentumvar.cryptarum (from the lectotype of P.succulentumvar.longifoliumfo.splendens, *W. Mönkemeyer*, JE 04004213!).

Plants medium-sized to large, usually yellowish gold, golden green, golden, very glossy; stems to 3 cm long; leaves spreading, in dry condition not shrunken, complanate, symmetrical, ovate, 2.50–3.00 × 0.80–1.40 mm (Fig. 5A); apex acuminate and not denticulate; laminal cells 130–240 × 10–18 μm at mid-leaf (Fig. 5D), cell areolation quite loose; decurrencies of 2–3 rows of rectangular cells; capsule inclined.

###### Distribution.

Asia (China, Democratic People’s Republic of Korea, Georgia, Islamic Republic of Iran, Republic of Korea, Russian Federation, Turkey); Europe (Albania, Andorra, Austria, Belarus, Belgium, Bosnia and Herzegovina, Bulgaria, Czech Republic, Denmark, Estonia, Finland, France, Germany, Greece, Hungary, Iceland, Ireland, Italy, Kosovo, Latvia, Lithuania, Luxembourg, Montenegro, Netherlands, North Macedonia, Norway, Poland, Portugal, Romania, Serbia, Slovakia, Slovenia, Spain, Sweden, Switzerland, Ukraine, United Kingdom); North America (Canada and U.S.A.).

##### ﻿ Plagiothecium succulentum var. propaguliferum

Taxon classificationPlantaeHypnalesPlagiotheciaceae

(E.Bauer) G.J.Wolski com. nov.

20892B99-3E0E-5D70-9A2E-53D43486C667

≡ Plagiotheciumsucculentumfo.propaguliferum E.Bauer, Deutsche Botanische Monatsschrift 20: 2. 1902. Lectotype (designated here): [Germany,] an Erlenstöcken in Erlbruche am Schiessniger Teiche bei B. Leipa, 250 m, ü. d. adr. M. *V. Schiffner*, *Bryotheca Bohemica 259*, 7 Aug. 1900, C-M-9395!

###### Description.

Plants medium-sized, dark golden to brown, very glossy; stems to 2.0–2.5 cm long; leaves spreading, in dry condition shrunken, complanate, symmetrical, ovate-lanceolate, 3.0–3.60 × 1.40–1.60 mm (Fig. 5B); apex acuminate, not denticulate; laminal cells linear-rhomboidal, linear-hexagonal, 180–260 × 17.0–20.0 μm at mid-leaf, (Fig. 5E), cell areolation loose; decurrencies of 2–3 rows of rectangular cells; capsule inclined.

###### Distribution.

Europe (Austria, Czech Republic, Denmark, Germany, Latvia); North America (Canada, U.S.A.).

##### ﻿ Plagiothecium succulentum var. cryptarum

Taxon classificationPlantaeHypnalesPlagiotheciaceae

(Renauld & Hérib. in Héribaud) G.J.Wolski comb. nov.

5BE9CAC8-02BF-5AFA-BD65-F219D5F8CF45

≡ Plagiotheciumdenticulatumvar.cryptarum Renauld & Hérib. *in* Héribaud, Mémoires de l’Académie des Sciences, Belles-lettres et Arts de Clermont-Ferrand, Deuxième Série 14: 229, 1899 ≡ Plagiotheciumsylvaticumvar.cryptarum (Renauld & Hérib.) P.Syd., Botanischer Jahresbericht 27(1): 200. 1904 ≡ Plagiotheciumroeseanumfo.cryptarum (Renauld & Hérib.) Jedl., Spisy Vydávané Přírodovĕdeckou Fakultou Masarykovy University 308: 37. 1948. Type: [France,] Central, près I’hôtel de Cournillou, Vallée de la Rue, sur le sol d’une grotte, Aug. 1894 & 1895, *J. Héribaud*. Lectotype (designated here): PC 0132577! Isolectotypes: PC 0132578!, PC 0132579!, PC 0132580!, PC 0132581!, PC 0132582!, PC 0132586!
= Plagiotheciumsucculentumvar.longifoliumfo.splendens Mönk., Die Laubmoose Europas 863. 1927. Lectotype (designated here): [Germany.] Kreuzenberg, bei Nieder Feer. Juli 1908, *W. Mönkemeyer*, JE 04004213! syn. nov.

###### Description.

Plants medium-sized to large, dark golden to golden brown; stems to 3–5 cm long; leaves spreading, not overlapping, in dry condition not shrunken, complanate, symmetrical or almost symmetrical, lanceolate, 1.9–3.5 × 0.6–1.0 mm (Fig. 5C); apex acuminate, filiform, and not denticulate; laminal cells 150–260 × 16–22 μm at mid-leaf (Fig. 5F), cell areolation loose; decurrencies of 2–3 rows of rectangular cells; capsule unknown so far.

###### Distribution.

Europe (France, Germany), but the range of this taxon still requires research.

##### ﻿ Plagiothecium cavifolium

Taxon classificationPlantaeHypnalesPlagiotheciaceae

(Brid.) Z.Iwats., Journal of the Hattori Botanical Laboratory 33: 360. 1970.

7244D2F5-9DDC-53A6-86CE-84CB3FB0E3EE

≡ Hypnum (Stereodon) cavifolium Brid., Bryologia Universa 2: 556. 1827 ≡ Stereodoncavifolius (Brid.) Brid., Bryologia Universa 2: 824. 1827. Type: [Canada,] in terra habitat in insula Terre Neuve, *La Pylaie*, B-Brid 915!
= Plagiotheciumroeseanum Hampe *ex* Schimp., Bryologia Europea 5: 193, 504, table X. 1851 ≡ Hypnumroeseanum Hampe *in* Bruch, Schimper and W.Gümbel, Bryologia Europea 5: 193, 504. 1851, nom. inval. ≡ Plagiotheciumsylvaticumvar.roeseanum (Hampe *ex* Schimp.) A.W.H.Walther & Moldendo, Die Laubmoose Oberfrankens 177. 1868 ≡ Plagiotheciumdenticulatumvar.roeseanum (Hampe *ex* Schimp.) Hérib., Mémoires de l’Académie des Sciences, Belles-lettres et Arts de Clermont-Ferrand, Deuxième Série, 14: 228. 1899 ≡ Plagiotheciumdenticulatumsubsp.roeseanum (Hampe *ex* Schimp.) Grout, Moss Flora of North America 3: 158. 1932. Type: [Germany,] Ad terram arenosam sub *Fagis* in monte Inselberg Thuringiae cl. *A. Roese* legit atque nobiscum benevole communicavit, JE 04004196!, JE 04004197!, JE 04004198!, JE 04004199!, HBG-021130!
= Plagiotheciumorthocladium Schimp., Bryologia Europea 5: 193, 504, table X. 1851 ≡ Plagiotheciumsylvaticumvar.orthocladium (Schimp.) Schimp., Corollarium Bryologiae Europaeae 115. 1856 ≡ Hypnumsylvaticumvar.orthocladium (Schimp.) Husn., Flore Analytique et Descriptive des Mousses du Nord-Ouest, 2 Edition 149. 1882 ≡ Plagiotheciumroeseanumvar.orthocladium (Schimp.) Limpr., Die Laubmoose Deutschlands, Oesterreichs und der Schweiz 3: 262. 1897 ≡ Plagiotheciumdenticulatumvar.orthocladium (Schimp.) Hérib., Mémoires de l’Académie des Sciences, Belles-lettres et Arts de Clermont-Ferrand, Deuxième Série, 14: 229. 1899 ≡ Plagiotheciumsylvaticumfo.orthocladium (Schimp.) Barkman, Phytosociology and Ecology of Cryptogamic Epiphytes 619. 1958, *comb. inval.* ≡ Plagiotheciumcavifoliumvar.orthocladium (Schimp.) Z.Iwats., Journal of the Hattori Botanical Laboratory 33: 371. 1970. Type: In m. Donnersberg Vogesi inferioris, *Th. Gumbel* legit auno 1842 (*n.v*.).
= Plagiotheciumattenuatirameum Kindb., Catalogue of Canadian Plants, Part VI, Musci 277. 1892 ≡ Plagiotheciumlaetumsubsp.attenuatirameum (Kindb.) Kindb., Canadian Record of Science 6(2): 72. 1894. Type: Canada, Québec, Chelsea in Gilmour’s Park, on rock, *J. Macoun 417*, 6 September 1889, herb. *I.Thériot*, PC0132687!
= Plagiotheciumroeseanumvar.angustirete Warnst., Verhandlungen des Botanischen Vereins der Provinz Brandenburg 42: 214. 1900 ≡ Plagiotheciumroeseanumfo.angistirete (Warnst.) Jedl., Spisy Vydávané Přírodovĕdeckou Fakultou Masarykovy University 308: 39. 1948. Type: Germany, Brandenburg, Chorin (Mark), Hohlweg am Bach, am Waldhohlwege im „Forstarten” mit Eurhynchiumschleicheri, *L. Loeske*, 10 Sep. 1899, herb. *H. Dohl*, JE 4004200!
= Plagiotheciumroeseanumvar.japonicum Cardot, Bulletin de la Société Botanique de Genève, sér. 2, 4: 385. 1912. Type: Japan, Aomori Pref., *Faurie 408* (“P.silvaticumvar.orthocladum Sch.”), herb. *J. Cardot*, PC 0132574!; idem, *Faurie 418*; Kanita, *Faurie 1812*; Hirosaki, *Faurie 1878*; Osorezan, *Faurie 2104*; château d’Akita, *Faurie 2904*; Nayoro, *Faurie 3078* in parte; Sambongi, *Faurie 3190*; Otaru, *Faurie 3753*; Tobetsu, *Faurie 3761*, KYO.

###### Description.

**Figure 6.**
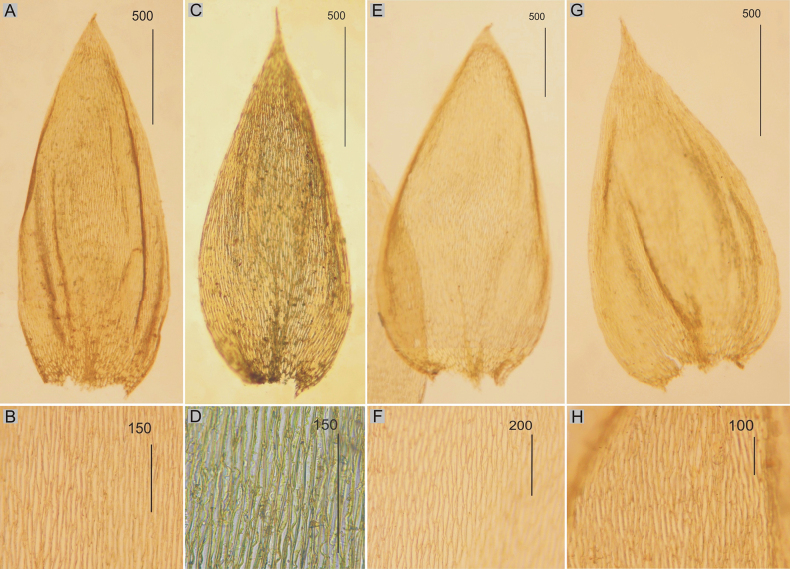
Selected, most important taxonomic features of taxa from the *Plagiotheciumcavifolium* complex **A**, **C**, **E**, **G** shape and dimensions of the leaves **B**, **D**, **F**, **H** shape and dimensions of cells from the middle part of the leaves **A, B***P.cavifolium* (from type of *Hypnumroeseanum*, *A. Roese*, JE4004197!) **C, D***P.ikegamii* (from type of *P.propaguliferum*, *Y. Iishiba*, PC 0132610!) **E, F***P.subjulaceum* (from type of P.roeseanumfo.umbrosa, *R. Schmidt*, HBG 021131!) **G, H***P.flaccidum* (from type of *Leskeaflaccida*, *J. Torrey*, B 31076701!), based on Wolski et al. 2022b changed.

Plants small-sized, yellowish-green to light green; stems 2–4 cm long; leaves julaceous, concave, imbricate, symmetrical, more or less folded, 1.2–2.5 × 0.6–1.0 mm (Fig. 6A); the apex not denticulate; laminal cells 100–150 × 10–12 μm at midleaf (Fig. 6B), cell areolation quite loose; decurrencies of 2–3 rows of rectangular to quadrate cells; setae 1.8–2.5 cm; capsule inclined.

###### Distribution.

Europe (Czech Republic, Denmark, Finland, Germany, Italy, Lithuania, Norway, Poland, Portugal, Romania, Serbia, Slovakia, Slovenia, Sweden, Switzerland, Ukraine, United Kingdom).

##### ﻿ Plagiothecium ikegamii

Taxon classificationPlantaeHypnalesPlagiotheciaceae

Sakurai, Botanical Magazine (Tokyo) 62: 113, f. 3. 1949.

B5D5B065-F871-5985-A7FD-C89112AF0649

= Plagiotheciumroeseanumvar.alpinum Kern, Jahresbericht der Schlesischen Gesellschaft für Vaterländische Cultur 91(2b): 64. 1914 ≡ Plagiotheciumroeseanumfo.alpinum (Kern) Jedl., Spisy Vydávané Přírodovĕdeckou Fakultou Masarykovy University 308: 37. 1948 ≡ Plagiotheciumalpinum (Kern) Jedl., Spisy Vydávané Přírodovĕdeckou Fakultou Masarykovy University 318: 5, 1950. Type: Italy, Felsritzen des Cruschettapasses an der Schweizer Grenze, 2300 m, 30 July 1913, *F. Kern*, PC 0132603!
= Plagiotheciumroeseanumfo.rigidum Jedl., Spisy Vydávané Přírodovĕdeckou Fakultou Masarykovy University 308: 37. 1948. Type (authentic specimens cited in Jedlička 1961): Moravia, Jeseníky, Švýcárna, 1300 m, ster., *J. Podpěra*, H.M.B.; Brno, Bílovice, cfr., *K. Doležal*, H.U.B., as. P.denticulatum; Adamov, in conc. riv. Kateřinský, ster., *J. Jedlička*, H.J.; Slovakia, Vysoké Tatry, Štrbské Solisko, in Calamagrostidetovillosae, solo granitico, 1385 m, ster., Krajina, H.U.P., sub P.denticulatum (*n.v*.).
= Plagiotheciumroeseanumfo.subdentatum Jedl., Spisy Vydávané Přírodovĕdeckou Fakultou Masarykovy University 308: 38. 1948 ≡ Plagiotheciumsubdentatum (Jedl.) Jedl., Spisy Vydávané Přírodovĕdeckou Fakultou Masarykovy University 318: 5. 1950. Type (authentic specimens cited in Jedlička 1961): Moravia, Jeseníky, ster. cum Desmatodon, Frank, H.P., Inter. p. Dalečín et Jimramov, 500 m, ster., *J. Podpěra*, H.P.; Carp. occid., Rožnov, s.m. Radhošt, versus Kluzov, ster., *J. Podpěra*, H.P.; Turcia, Salonichi, Kartaš-dagh, 1200 m, ster., *J. Podpěra*, H.P. (*n.v*.).
= Plagiotheciumpropaguliferum Broth., in sched. Basis: Japan, Sendai, *Y. Iishiba*, July 1907, herb. *J. Cardot*, *I. Thériot*, PC 0132610!
= Plagiotheciumapiculatum Sakurai, in sched. Basis: Japan, Niigata Pref., Toyanao, 2 Apr. 1942, *Y. Ikegami 4256*, MAK B115140!

###### Type.

Japan, Etigo Prov., Mt. Renge, ad terram, ca. 2200 m, *Y.Ikegami 11270*, herb. *K. Sakurai 16336*, August 1949; Shinano Prov., Mt. Shirouma, 2500 m, *N. Takaki in herb*. *K. Sakurai 16368*, August 1949 (*n.v*.).

###### Description.

Plants medium-sized, yellowish-green; stems 2–4 cm long; leaves julaceous, concave, imbricate, symmetrical, more or less folded, 1.3–2.0 × 0.5–1.2 mm (Fig. 6C); the apex denticulate; laminal cells 100–150 × 10–12 μm at midleaf (Fig. 6D), cell areolation quite loose; decurrencies of 1–2 rows of rectangular to quadrate cells; capsule inclined.

###### Distribution.

Asia (Japan); Europe (Czech Republic, Italy), but the range of this taxon still requires research.

##### ﻿ Plagiothecium subjulaceum

Taxon classificationPlantaeHypnalesPlagiotheciaceae

(Meyl.) Jedl., Spisy Vydávané Přírodovĕdeckou Fakultou Masarykovy University 318: 5. 1950.

2D25447E-3DC3-5D9E-986E-93CAA0531192

≡ Plagiotheciumroeseanumvar.subjulaceum Meyl. *in* J.J.Amann, Flore des Mousses de la Suisse 2: 328. 1918 ≡ Plagiotheciumroeseanumfo.subjulaceum (Meyl.) Jedl., Spisy Vydávané Přírodovĕdeckou Fakultou Masarykovy University 308: 38. 1948. Type: (authentic specimens cited in Jedlička 1961): Typus secundum specimina a *J. Podpěra* in Moravia orientali (Rajnochovice) collecta, descriptus est. ČSSR – Rapotice, ster. (*Doležal*, H.U.B.). Carp. occident.: Bašta pr. Rajnochovice, ad rup. arenac., ster. (*Podpěra*, H.M.B.). – Slovakia. Bratislava: in conv. Pajštúnska dolina, ster. (*Podpěra*, H.P.) (hygromorphosa).
= Plagiotheciumsylvaticumvar.cavifolium Jur. *in* Rabenhorst, Bryotheca Europaea 16: 765. 1864. Type: *Bryotheca europaea 765*, Auf nacktem Boden in Buchenwäldern auf Nagelfluhe am Mönchsberge bei Salzburg, Sauter (als. *Plag.Lucens Sauter* n. sp.), distrib. *L. Rabenhorst*, FH 220150, MO 406590, PC 00132571!
= Plagiotheciumsilvaticumvar.latifolium Röll, Deutsche Botanische Monatsschrift 9: 131. 1891, *non* Cardot, Bulletin de la Société Botanique de Genève, sér. 2, 4: 385. 1912, *hom. illeg*. ≡ Plagiotheciumsylvaticumvar.latifolium Röll, Hedwigia 56: 229. 1915, *hom. illeg*. Type: Germany, Thuringia, im Werrthal bei Plankenburg an der hohen Schlaufe bei Ilmenau, *J. Röll*, HBG 21134!
= Plagiotheciumroeseanumfo.umbrosa Mönk., Die Laubmoose Europas 863. 1927. Type: Germany, Thüringen, Finsteres Loch, *Rich Schmidt Lips*., 20 June 1916, HBG 021131!
= Plagiotheciumsucculentumvar.longifolium Mönk., Die Laubmoose Europas 863, f. 206b. 1927 ≡ Plagiotheciumsylvaticumfo.longifolium (Mönk.) C.E.O.Jensen, Skandinaviens Bladmossflora 495. 1939 ≡ Plagiotheciumsucculentumfo.longifolium (Mönk.) Jedl., Spisy Vydávané Přírodovĕdeckou Fakultou Masarykovy University 308: 42. 1948. Lectotype (designated by Wolski et al. 2022b): Germany, Thüringen Wald, am Simmetsberg im Ungeheuren Grund, Hess, Aug. 1872, JE 4004211! Isolectotype: Germany, Thüringen, Annathal bei Eisenach, Hess, Aug. 1872, JE 4004212!
= Plagiotheciumfujiyamae Sakurai, in sched. Basis: Japan, Aokigahara, Fuji, Yamanashi Pref., *T. Maede 1462*, 9 Nov. 1950, herb. *K.Sakurai*, MAK 57198!
= Plagiotheciumnakajimae Sakurai, in sched. Basis: Japan, Chichinu, Nagano, 6 Nov. 1951, herb. *K. Sakurai 761*, MAK B57158!

###### Description.

plants medium-sized, yellowish-green to green, stems 2–4 cm long; leaves julaceous, concave, imbricate, symmetrical, more or less folded, 1.3–2.6 × 0.6–1.2 mm (Fig. 6E); the apex acuminate, not denticulate; laminal cells 60–100 × 10–16 μm at midleaf (Fig. 6F), cell areolation quite loose; decurrencies of 2–3 rows of rectangular cells; capsule inclined.

###### Distribution.

Asia (Japan); Europe (Germany), but the range of this taxon still requires research.

##### ﻿ Plagiothecium flaccidum

Taxon classificationPlantaeHypnalesPlagiotheciaceae

(Brid.) G.J.Wolski & W.R.Buck, Diversity 14(8): 633. 2022.

C5A59F31-1017-5E1B-BB17-F6C3F79B05F1

≡ Leskeaflaccida Brid., Bryologia Universa 2: 308. 1827. Type: In Republica Massachusets Americae Foedewatae circa Noveboracum in rupis habitat, caespitosa, caespitum basi e congerie caulium veterarnorum marcescentium constante, *Torrey 67*, 1820, B 31076701!
= Hypnumsullivantiae Schimp. *ex* Sull., A Manual of the Botany of the Northern United States. Second Edition 680. 1856 ≡ Plagiotheciumsullivantiae (Schimp. *ex* Sull.) Schimp. *ex* A.Jaeger, Bericht über die Thätigkeit der St. Gallischen Naturwissenschaftlichen Gesellschaft 1876–77: 450. 1878 ≡ Plagiotheciumsylvaticumvar.sullivantiae (Schimp. *ex* Sull.) Renauld & Cardot, Revue Bryologique 20: 22. 1893. Type: Ohionis et Novae Angliae, in rupium fissuris terra impletis, Musci Boreali-Americani 355, PC 0132606!, PC 0132607!; idem herb. *M.Bizot 13157*, PC 0132608!
= Plagiotheciumroeseanumvar.orthocladonfo.propaguliferum Jedl., Spisy Vydávané Přírodovĕdeckou Fakultou Masarykovy University 308: 39. 1948, *hom. illeg*., *non* (R.Ruthe) Jaap, Verhandlungen des Naturwissenschaftlichen Vereins in Hamburg, ser. 3, 7: 36. 1900 ≡ Plagiotheciumroeseanumvar.orthocladonfo.moravicum Pilous *in* Jedlička, Spisy Přírodovĕdecké Fakulty University v Brnĕ 422: 214. 1961, nom. nov. Type: Moravia, conv. flum. Oslava, ster., Latzel, H.L., observavi (*n.v*.).

###### Description.

Plants small-sized, yellowish-green to light green; stems 2–3 cm long; leaves julaceous, concave, imbricate, symmetrical, more or less folded, 1.5–1.8 × 0.7–0.8 mm (Fig. 6G); the apex not denticulate; laminal cells 75–130 × 10–12 μm at midleaf (Fig. 6H), cell areolation quite loose; decurrencies of 1–2 rows of rectangular to quadrate cells; capsule erect.

###### Distribution.

Europe (Czech Republic); North America (U.S.A.), but the range of this taxon still requires research.

##### ﻿ Plagiothecium tenue

Taxon classificationPlantaeHypnalesPlagiotheciaceae

(Jedl.) G.J.Wolski and W.R.Buck, Divesity, 14(8): 633 [16]. 2022.

17145DF3-1D29-50C8-97DA-32E6FE67E814

≡ Plagiotheciumroeseanumfo.tenue Jedl., Spisy Vydávané Přírodovĕdeckou Fakultou Masarykovy University 308: 38. 1948. Type (authentic specimens cited in Jedlička 1961): Silesia, Cuidowa, Steinberg, ster. *Paul*, H.M.B.; Bohemia, Beroun, Skryje, in decl. Vosník col. ster., *Šmerda*, H.Š. (sub P.denticulatum); Moravia, Jeseníky, Quarklöcher, pr. Brummlitz, ster. una cum *Barbula rigida* et *Fissidens pusillus*, Latzel, H.L.; Voskovice, in silva umbrosa pr. oppid, 300 m, ster., *Doležal*, H.P.; Brno, Kuřím, ad col. Baba, ster. *Doležal*, H.M.B. (sub P.denticulatum); Kůňku pr. Obora, str., *Podpěra*, H.P.; Mor. Krumlov, ad rup. perm., 300 m, ster. *Podpěra*, H.M.B.; Carp. occid., in m. Ondřejník, pr. Frýdlant, ster., *Podpěra* H.P.; in m. Lysá in conv. riv. Mazák, ster., *Podpěra*, H.P.; Rajnochovice, Pomsko, ster., *Podpěra*, H.P.; Rychtářov, in conv., V. Haná, ster., *Podpěra*, H.P.; Unčov, cataract. Řešovský, ster., *Podpěra*, H.P. Austria. Koralpe, Theisseneppergraben, solo granit., 800 m, ster., Latzel, H.L.; Pressinggraben, ster. Latzel, H.L. (s. *P. Roeseanum gracile*). Jugoslavia, Surdulica, in conv. Vrla reka, ster. *Podpěra*, H.P.; Vrane-Kazandžol, ster., Podpěra, H.P (*n.v*.).
= Plagiotheciumroeseanumfo.tenuesubfo.propaguliferum Jedl., Spisy Vydávané Přírodovĕdeckou Fakultou Masarykovy University 308: 38. 1948, *hom. illeg*. ≡ Plagiotheciumroeseanumsubfo.gemmicladum Pilous, Spisy Přírodovĕdecké Fakulty University v Brnĕ 422: 212. 1961. Type (authentic specimens cited in Jedlička 1961): Suecia, Skåne, Bokeberg, ster., *Möller*, H.M.B.; Germania, Sachsen, Plauen, ad saxa umber. in conv. Elstertal, ster., *Stolle*, H.P. (planta pulcherima!!); Austria, Saualpe, Pöllinggraben, cfr., Latzel, H.L.; Wien, ad arcem Greifenstein, 300 m, cfr., Baumgartner, Krypyog. exsicc. M.N. no. *1788a*, H.M.P.; Bohemia, Praha, Hasenburg, 250 m, ster., Bauer; Musc. eur. exsicc. no. *1311*, H.P., H.M.B., H.M.P., H.U.B. (sub *P. Roeseanum* fo. *gracilescens*) *Bauer in sched*.; Řevnice, ster. *Podpěra*, H.P. (sub P.denticulatum); Nové Mešto n. Met. ad rup. fyllit. Peklo, ster., *Šmaeda*, H.Š.; Berno, Skryje, ster., cum *Anomodon attenuatus* et *Mnium cuspidatum*, *Šmaeda*, H.Š. (sub P.denticulatumpropaguliferum); Tusset, 1000 m, ster., *Podpěra*, H.P. (sub P.denticulatum); Moravia, Jeseníky, Švýcárna, ster. 1300 m, *Podpěra*, H.P.; Hokšár, ster., *Podpěra*, H.P.; Brno, pr. arcem Veveří, ster., *Podpěra*, H.P.; in conv. Bílý potok, sup. Hluboké, ster. *Podpěra*, H.P. (sub *P. Roeseanum umbrosum*); Adamov, in conv. riv., Josefovský, ster., *Podpěra*, H.P.; in conv. rivuli Kateřinský potok, ster., *J.Müller*, H.U.B.; ad rup. syenit. in conv. flum. Svitava, inter Adamov et Blansko, ster., *Podpěra*, H.P.; Rousínov, Vítocický žleb, *Podpěra*, H.P. (sub *P. Roeseanum gracile fo. tenullum*) Podpěra in sched.; Mor. Krumlov, ad rup. perm., 300 m, ster., *Podpěra*, H.P.; Carp. occid., ad ped. m. Lysá Hora, pr. Staré Hamry, ster., *Podpěra*, H.P.; in m. Hostýn, ster., *Podpěra*, H.P. (*n.v*.).
= Plagiotheciumroeseanumfo.acuminatum Jedl., Spisy Vydávané Přírodovĕdeckou Fakultou Masarykovy University 308: 40. 1948 ≡ Plagiotheciumcavifoliumfo.acuminatum (Jedl.) Z.Iwats., Journal of the Hattori Botanical Laboratory 33: 363. 1970. Type (authentic specimens cited in Jedlička 1961): Austria, Arlingsgraben, ster., Latzel, H.L. Bohemia, Praha, ad rup. lydit., 200 m, ster., *Šmarda*, H.Š.; Babka pr. Řevnive, 400 m, Bauer, Bryoth. Bohem. no *255*, H.U.P., H.Š., H.M.P. (sub P.roeseanumtypicum); Mladá Boleslav, in conv. Choboty, cfr., *Podpěra*, H.P., Moravia, Jeseníky, Dolní Lipová, ster., Latzel, H.L.; in conv. riv. Seifen pr. Vernířovice, 800 m, ster., *Podpěra*, H.P.; Znajmo, Eisleiten pr. Varanoc, ster., *Podpěra*, H.P.; Senohrady, ad rup., ster., *Podpěra*, H.P.; Unčov, ad cataract. Řešovský, 400 m, ster., *Podpěra*, H.P.; Slovakia, Babia Góra, ad lignus putr., ster., *Šmerda*, H.Š. (sub P.silvaticumlongifolium); Bielské Tatry, in conv. Havran, 1100 m, cum Blepharostomatrichophyllum, ster., *Šmerda*, H.Š (*n.v*.).

###### Description.

**Figure 7.**
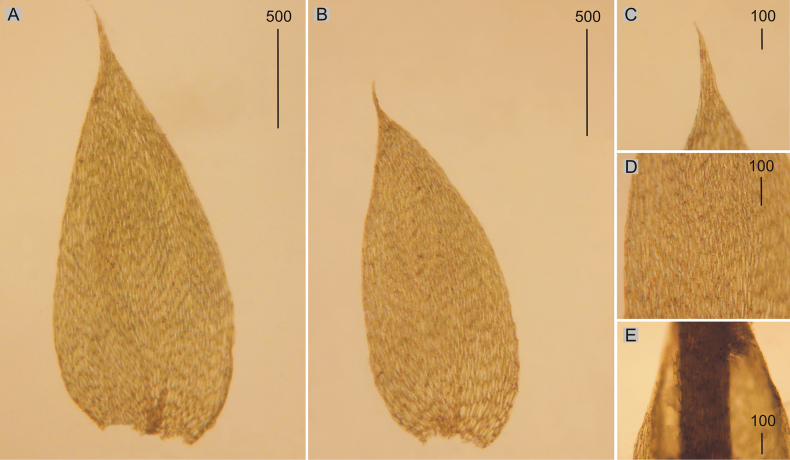
Selected, most important taxonomic features of taxa from the *Plagiotheciumcavifolium* complex **A, B** shape and dimensions of the leaves **C** leaves apex **D** shape and dimensions of cells from the middle part of the leaves **E** decurrencies on the stem. **A–E***Plagiotheciumtenue* (from P.roeseanumfo.tenue, herb. *A. Baros*, det. *J. Jedlička*, BRNU 592!).

Plants small, yellowish-green to light green; stems 0.5–1.5 cm; leaves not julaceous; flat, not imbricate, asymmetrical, ovato-lanceolate, 1.2–1.8 × 0.6–0.8 mm (Fig. 7A, B); the apex acuminate, long (Fig. 7C), not denticulate; laminal cells 70–100 × 10–12 μm at midleaf (Fig. 7D), cell areolation quite loose; decurrencies of 2–3 rows of rectangular cells; capsule inclined.

###### Distribution.

Europe (Austria, Czech Republic, Germany, Poland, Serbia, Slovakia, Sweden), but the range of this taxon still requires research.

#### Sect. Leptophyllum Jedl.

##### ﻿ Plagiothecium berggrenianum

Taxon classificationPlantaeHypnalesPlagiotheciaceae

Frisvoll, Lindbergia 7: 96, f. 2: a–i. 1981.

FCBBB5DB-4B65-5C56-901E-1641029E1C4A

###### Type material.

Holotype: Norway, Svalbard, Haakonvii Land, Krossfjorden, Kollerfjorden, below bird cliff in Christian Michelsenfjell W, 50 m, 22 July 1974, *A. A. Frisvoll*, TRH B-19507! Isotype: C-M-20077! Paratypes: Lilliehöökfjorden, bird cliff in Nilsfjellet N, 50 m, 22 July 1974 (TRH); Bellsund, Vårsolbukta, by Camp Miller, 25 m, 29 July 1980, *Olsen*; S of Ingeborgfjellet, 10 m, 13 July 1980, *Olsen* (TRH); Sjuøyane; Parryya, 80°40'N, below brid cliff, 1868, *Berggren*, TRH.

###### Description.

**Figure 8.**
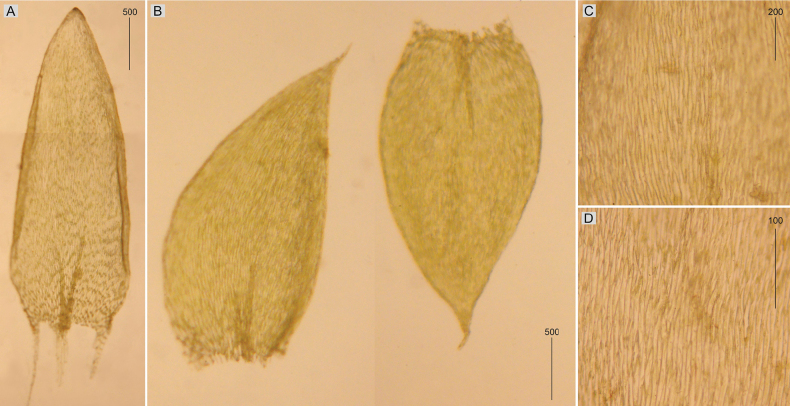
Selected, most important taxonomic features of *Plagiotheciumberggrenianum* and *Plagiotheciumsvalbardense***A, B** shape and dimensions of the leaves **C, D** shape and dimensions of cells from the middle part of the leaves **A**, **C***P.berggrenianum* (from holotype, *A. A. Frisvoll*, TRH B-19507!) **B**, **D***P.svalbardense* (from holotype, *A. A. Frisvoll*, TRH B-19481!).

Plants small, dense, yellowish green to green, glossy, with metallic luster; stems erect, 3–9 cm long; leaves very crowded on stem, julaceous, imbricate, symmetrical and very concave, thus the leaves often cracked, plicate, 1.5–3.1 × 0.7–1.1 mm (Fig. 8A); the apex acuminate, recurved, hook-shaped; margins denticulate or not near the apex; laminal cells 120–170 × 12–15 μm at midleaf (Fig. 8C), cell areolation quite loose; decurrencies well developed, consisting of 3–4 rows of rectangular cells.

###### Distribution.

Asia (Russian Federation); Europe (Norway); North America (Canada, U.S.A.).

##### ﻿ Plagiothecium svalbardense

Taxon classificationPlantaeHypnalesPlagiotheciaceae

Frisvoll, Norsk Polarinstitutt Skrifter, Part 2. Bryophytes 198: 103. 1996.

FC978C6E-891B-5D57-9558-1439A5FC9901

###### Type material.

Holotype: Norway, Svalbard, Krossfjorden, Kollerfjorden, below a bird cliff in Christian Michelsenfjella W, 50 m, 22 July 1974, *A. A. Frisvoll*, TRH B-19481! Isotypes: O, S, TRH.

###### Description.

Plants medium-sized, dark green, dull, without metallic luster; stems 2–4 cm long, more or less julaceous; leaves concave, two types of leaves: symmetrical and asymmetrical, ovate, 2.4–2.8 × 1.2–1.5 mm (Fig. 8B); the apex acuminate, often gently curved; margins not denticulate near the apex; laminal cells narrowly elongate-hexagonal, asymmetric, 80–120 × 7–10 μm at midleaf (Fig. 8D), cell areolation tight; decurrencies of 3 rows of rectangular to quadrate cells.

###### Distribution.

Asia (Russian Federation); Europe (Norway, Sweden).

##### ﻿ Plagiothecium curvifolium var. curvifolium

Taxon classificationPlantaeHypnalesPlagiotheciaceae

Schlieph. ex Limpr., Die Laubmoose Deutschlands, Oesterreichs und der Schweiz 3: 269. 1897.

9AFF3128-7259-546F-8EE1-914A929C5F09

###### Type material.

Lectotype (designated by Wolski et al. 2022a): Germany, Thuringia, in feuchten Nadelwäldern, Schmücke, 29 July 1880, *D. K. Schliephacke*, JE 04004091! Isolectotypes: HBG 02115!, PC 01322640!, WRSL!, G!, DUKE 155945.

###### Description.

**Figure 9.**
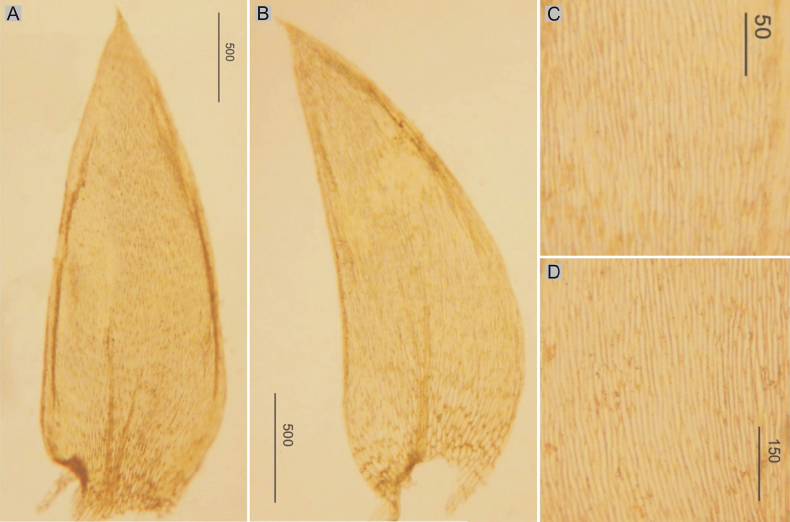
Selected, most important taxonomic features of taxa from the *Plagiotheciumcurvifolium* complex **A, B** shape and dimensions of the leaves **C, D** shape and dimensions of cells from the middle part of the leaves **A**, **C**P.curvifoliumvar.curvifolium (from lectotype of *P.curvifolium*, *K. Schliephacke*, JE 04004091!) **B**, **D**P.curvifoliumvar.recurvum (from lectotype of P.denticulatumvar.recurvum, *C. Warnstorf*, JE 04004201!), based on Wolski et al. 2022a changed.

Plants medium-sized, yellow-green to green; stems 1.5–2.5 cm long, complanate-foliate; leaves symmetrical or almost symmetrical, gently imbricate, lanceolate to ovate-lanceolate, concave, slightly curved towards the ground, 1.7–2.7 × 0.7–1.5 mm (Fig. 9A); margin incurved, delicately on both sides or strongly on one side; the apex acuminate, not denticulate; laminal cells linear-vermicular, 110–155 × 8–9 μm at midleaf (Fig. 9C), cell areolation tight; decurrencies of 2–3 rows of rectangular cells forming semi-distinct auricles, some cells from external row inflated; capsules inclined to horizontal.

###### Distribution.

Asia (Georgia, Russia); Europe (Belgium, Czech Republic, Denmark, Estonia, Finland, France, Germany, Great Britain, Hungary, Latvia, Netherlands, Poland, Romania, Spain, Sweden); North America (Canada, U.S.A.).

##### ﻿ Plagiothecium curvifolium var. recurvum

Taxon classificationPlantaeHypnalesPlagiotheciaceae

(Warnst.) G.J.Wolski & W.R.Buck, PLoS ONE 17(11): e0275665. 2020.

3B72CAD8-A89B-5A6A-86A8-9E3E0786D105

≡ Plagiotheciumdenticulatumvar.recurvum Warnst., Verhandlungen des Botanischen Vereins für die Provinz Brandenburg und die Angrenzenden Länder 27: 73. 1885. Lectotype (designated by Wolski et al. 2022a): Germany, prov. Brandenburg, auf nacktem Bodem in Kiefernschonungen vor Altruppin, Neuruppin, *C. Warnstorf*, JE 04004201! Isolectotypes: G!
= Plagiotheciumcurvifoliumvar.hypnophyllum Ukrainskaya, Novosti Sistematiki Nizaikh Rastenii 31: 183, f. 12–14. 1996. Type: [Russia,] Prov. Mosquensis, distr. Krasnogorskensis, 2 km ad austro-occidentem a Krasnogorsk. Ad Betulam in silva, 28 VII 1986, *Ignatov*. In herbario bryologico Horti Botanici Publici Mosquae conservatur, MHA, VLA!

###### Description.

Plants medium-sized, bright-green to green; stems 1.5–2.0 cm long; leaves complanate, strongly asymmetrical, hooked, lanceolate, concave, curved towards the ground, 1.7–2.2 × 0.6–0.9 mm (Fig. 9B); margin sometimes incurved; the apex acuminate, usually denticulate by 2–3 teeth; cells linear-vermicular, 60–120 × 7–9 μm at midleaf (Fig. 9D), cell areolation tight; decurrencies forming semi-distinct auricles, of 2–3 rows of rectangular, sometimes inflated cells; capsules inclined.

###### Distribution.

Asia (Russia); Europe (Austria, Belgium, Czech Republic, Denmark, Finland, France, Germany, Great Britain, Hungary, Latvia, Poland, Slovakia, Sweden); North America (Canada).

##### ﻿ Plagiothecium decursivifolium

Taxon classificationPlantaeHypnalesPlagiotheciaceae

Kindb. in Macoun & Kindberg, Catalogue of Canadian Plants, Part VI, Musci 277. 1892.

AF3BED5A-6E7B-5001-A8C8-B714BD77C1AA

= Plagiotheciumcurvifoliumfo.julaceum Culm. *in* E.Bauer, Musci Europaei Exsiccati 27: 1307. 1915. Lectotype (designated by Wolski et al. 2022a): Switzerland, auf Tannenwurzeln ini der Nähe der oberen Waldgrenze, Burgfeld ob Beatenberg, Kanton Bern, 1630–1700 m, 31 July 1912, *Musci eur.exs. 1307*, *P. Culman*, C-M-9120! Isolectotype: MO 3974490!

###### Type material.

Lectotype (designated by Wolski et al. 2022a): Canada, Ontario, Belleville, on cedar (*Thujaoccidentalis*) stump in a swamp, 5 miles west of Belleville, Ont. *J. Macoun*, *N. C. Kindberg*, PC 0132686! Kindberg Canadian types should be at S with duplicates at CANM

###### Description.

**Figure 10.**
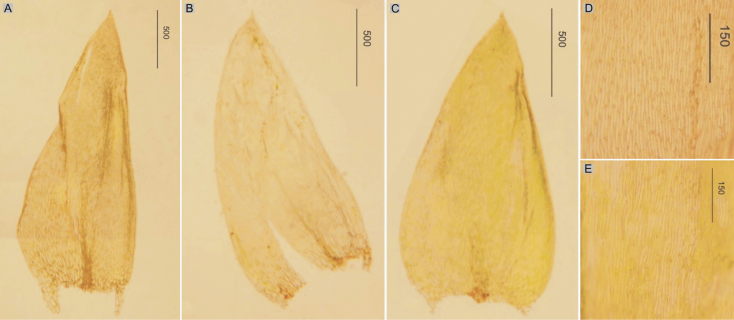
Selected, most important taxonomic features of taxa from the *Plagiotheciumcurvifolium* complex **A–C** shape and dimensions of the leaves **D, E** shape and dimensions of cells from the middle part of the leaves **A**, **D***P.decursivifolium* (from lectotype, *P. Culmann*, C-M-9120!) **B, C**, **E***P.imbricatum* (from holotype, *G. J. Wolski*, LOD 15015!), based on Wolski et al. 2022a changed.

Plants medium-sized to small, yellow to yellow-green; stems 0.5–1.5 cm long; leaves gently julaceous and imbricate, folded, ovate to ovate-lanceolate, asymmetrical, concave, often cracked at the base, 1.3–2.5 × 0.4–1.8 mm (Fig. 10A); the apex acuminate, not denticulate or rarely with one tooth; cells linear-vermicular, 95–190 × 6–10 μm at midleaf (Fig. 10D), cell areolation tight; decurrencies of 3–5 rows of rectangular, quadrate, often inflated cells forming semi distinct auricles.

###### Distribution.

Asia (China); Europe (Austria, Belgium, Czech Republic, Denmark, Finland, France, Germany, Hungary, Latvia, Netherlands, Poland, Slovakia, Sweden, Switzerland); North America (Canada).

##### ﻿ Plagiothecium imbricatum

Taxon classificationPlantaeHypnalesPlagiotheciaceae

G.J.Wolski & W.R.Buck, PLoS ONE, 17(11): e0275665. 2020.

3EAC9F16-CA16-5C16-9E30-6DFB30EC5C8F

###### Type material.

Holotype: Poland, kujawsko-pomorskie Voivodeship, surroundings of Dolina rzeki Brdy reserve, slope near the river on soil in mixed forest, 13 July 2020, *G. J. Wolski 424*, LOD 15015! Isotypes: NY 04688394!, SZUB-B 00001!

###### Description.

Plants small, bright-green to green; stems 0.7–1.5 cm long, densely foliate; leaves julaceous and imbricate, two types of leaves: symmetrical and asymmetrical, the symmetrical ones: folded, lanceolate, concave, sometimes strongly cracked at the base, asymmetrical ones: ovate, slightly concave or flat, both types of leaves identical in size, 1.2–2.3 × 0.7–1.0 mm (Fig. 10B, C); the apex acuminate, not denticulate; cells linear-vermicular, 80–190 × 5–9 μm at midleaf (Fig. 10E), cell areolation tight; decurrencies of 3–4 rows of rectangular, quadrate often inflated cells forming semi distinct auricles; capsules unknown so far.

###### Distribution.

Europe (Great Britain, Netherlands, Poland); North America (Canada).

##### ﻿ Plagiothecium laetum var. laetum

Taxon classificationPlantaeHypnalesPlagiotheciaceae

Schimp., Bryologia Europea 5: 184, 495, Tab. II. 1851.

0F6ADC9C-60E5-56B4-8B33-BCB271C5F113

≡ Leskealaeta (Schimp.) Berggr., Acta Universitatis Lundensis, 2 Afd., 3(7): 8. 1866 = Plagiotheciumdenticulatumvar.laetum (Schimp.) Lindb., Animadversiones de Hypno elegante 31. 1867 ≡ Plagiotheciumdenticulatumsubsp.laetum (Schimp.) Kindb., Bihang till Kongliga Svenska Vetenskaps-Akademiens Handlingar 7(9): 46. 1883 ≡ Hypnumdenticulatumvar.laetum (Schimp.) Lindb. *in* Lesquereux & James, Manual of the Mosses of North America 367. 1884. Type: [Switzerland,] in Rhaetic Alpe Albula, ubi in regione sylvatica versus Ponte in logno putrido, et supra hanc reionem prope Weissenstein, in rupium fissuris Dicrano gracilescenti intermixtum, *W. P. Schimper* aestate 1845 detexit. Nusquam alias adhuc observatum est. Syntype: PC 0132699!, PC0132701!

###### Description.

**Figure 11.**
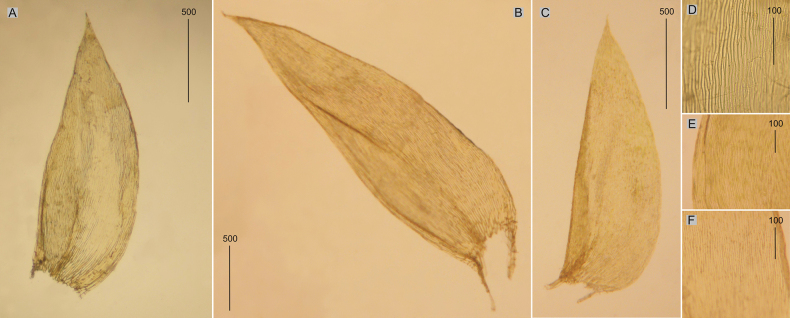
Selected, most important taxonomic features of taxa from the *Plagiotheciumlaetum* complex **A–C** shape and dimensions of the leaves **D–F** shape and dimensions of cells from the middle part of the leaves **A**, **D**P.laetumvar.laetum (from syntype, *W. P. Schimper*, PC 0132699!) **B**, **E**P.laetumvar.hercinicum (from lectotype of Plagiotheciumdenticulatumvar.hercynicum, *F. Gravet*, C-M-9387!) **C**, **F***P.rossicum* (from the original collection of *P.rossicum*, *M. S. Ignatov*, MHA9041632!).

Plants small, light green, glossy; leaves forming 20–70° angle with stem, complanate, more or less concave, asymmetrical, ovate-lanceolate, with one side almost flat, 1.0–2.0 × 1.1–1.3 mm (Fig. 11A); the apex acute, denticulate near the apex or not; laminal cells linear, 80–150 × 6–8 μm at midleaf (Fig. 11D), cell areolation dense; decurrencies of 1–3 rows of rectangular cells; setae 1.3–1.8 cm, capsule straight.

###### Distribution.

Asia (Azerbaijan, China, Democratic People’s Republic of Korea, Georgia, Islamic Republic of Iran, Japan, Kazakhstan, Kyrgystan, Mongolia, Republic of Korea, Russian Federation, Taiwan, Turkey); Europe (Albania, Andorra, Austria, Belarus, Belgium, Bosnia and Herzegovina, Bulgaria, Croatia, Czech Republic, Denmark, Estonia, Finland, France, Germany, Greece, Hungary, Ireland, Italy, Kosovo, Latvia, Lichtenstein, Lithuania, Luxemburg, Montenegro, Netherlands, Norway, Poland, Portugal, Romania, Serbia, Slovakia, Slovenia, Spain, Sweden, Switzerland, Ukraine, United Kingdom); North America (Canada, U.S.A.).

##### ﻿ Plagiothecium laetum var. hercinicum

Taxon classificationPlantaeHypnalesPlagiotheciaceae

(Jur. ex Grav.) G.J.Wolski comb. nov.

38B70026-7061-574A-92E5-0F84FE45A008

≡ Plagiotheciumdenticulatumvar.hercynicum Jur. *ex* Grav., Bulletin de la Société Botanique de Belgique 13: 430. 1874. Type: Belgium, Loutte-Saint-Pierre, sur les rochers ombragés et au pied des arbres dans les bois humides. Lectotype (designated here): Belgium, Loutte-Saint-Pierre, rochers ombragés, Oct. 1872, *F. Gravet*, C-M-9387!

###### Description.

Plants medium-sized, yellowish to yellowish golden; stems 1.5–2.0 cm long; leaves complanate, asymmetrical, lanceolate, concave, not curved towards the ground, 2.0–2.4 × 0.7–1.0 mm (Fig. 11B); margin incurved; the apex acuminate, denticulate by 2–3 teeth; cells linear-vermicular, 120–170 × 6–10 μm at midleaf (Fig. 11E), cell areolation tight; decurrencies of 2–3 rows of rectangular, quadrate cells; capsule straight.

###### Distribution.

Europe (Belgium), but the range of this taxon still requires research.

##### ﻿ Plagiothecium rossicum

Taxon classificationPlantaeHypnalesPlagiotheciaceae

Ignatov & Ignatova, Arctoa 28: 33. 2019.

9CBD38AE-BF45-5DF2-B513-D26BD56E780C

###### Type material.

Holotype: Russia, Pskov Province, Nevel’sk Distr., vicinities of Ustavnoe Settl. (near Yazno Lake), pine forest, at base of pine trunk, 26.IX.2001, Zolotov P504, MHA9041611.

###### Description.

Plants small, light green; stems 0.6–1 cm long; leaves forming 40–100° angle with stem, distinctly complanate, spreading, asymmetrical, ovate-lanceolate, 0.7–1.6 × 0.3–0.6 mm (Fig. 11C); the apex acute to acuminate; margins flat, denticulate or not near the apex; laminal cells narrow, 70–130 × 6–7 μm at midleaf (Fig. 11AF), cell areolation tight; decurrencies of 2–3 rows of rectangular cells; setae 1.0 cm, capsules more or less slightly inclined.

###### Distribution.

Asia (Russian Federation); Europe (Poland), but the range of this taxon still requires research.

#### Sect. Rectithecium (Hedenäs & Huttunen) J.T.Wynns

##### ﻿ Plagiothecium piliferum

Taxon classificationPlantaeHypnalesPlagiotheciaceae

(Sw.) Schimp., Bryologia Europea 5: 186, 496, Tab. III. 1851.

17341FE0-0497-558A-A2DD-71C374291EE0

≡ Leskeapilifera Sw. *in* C.J.Hartman, Handbok i Skandinaviens Flora 419. 1820 ≡ Hypnumdenticulatumvar.piliferum (Sw.) Wahlenb., Flora Suecica (Wahlenberg) 2: 710. 1826 ≡ Neckerapilifera (Sw.) Spruce, Musci Pyrenaici 66. 1847 ≡ Isopterygiumpiliferum (Sw.) Loeske, Studien zur Vergleichenden Morphologie und Phylogenetischen Systematik der Laubmoose 169. 1910 ≡ Plagiotheciellapilifera (Sw.) M.Fleisch. *in* Brotherus, Die natürlichen Pflanzenfamilien, Zweite Auflage, 11: 466. 1925 ≡ Dolichothecapilifera (Sw.) M.Fleisch. *ex* Podp., Conspectus Muscorum Europaeorum 683. 1954 ≡ Rectitheciumpiliferum (Sw.) Hedenäs & Huttunen, Botanical Journal of the Linnean Society 171(2): 344. 2013. Type: In rupe praerupta cujus totam parietem verticalem obducit horti regalis Haga-Park prope Holmiam cl. Swartz detexit ibidemque serius legerunt *Lindberg*, *Thedenius*, *Angström*, *W. P. Sch.*, e.a; ex Ostrogothiae monte Halberget cl. Holmgren, e Pyrenaeorum umbrosissima valle *de Jéret* ubi ad latera scopulorum graniticorum terram versus spectantia laete viget cl. *R.Spruce* misit.

###### Description.

**Figure 12.**
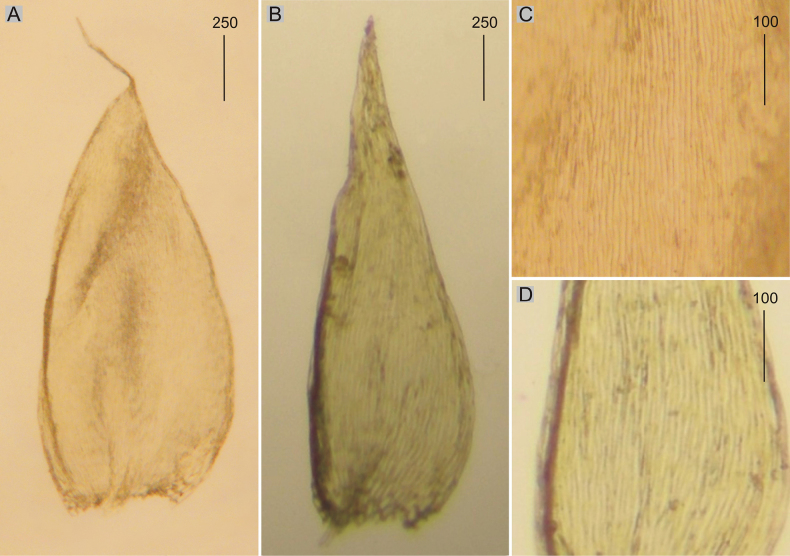
Selected, most important taxonomic features of the *Plagiotheciumpiliferum* and *Plagiotheciumlatebricola***A, B** shape and dimensions of the leaves **C, D** shape and dimensions of cells from the middle part of the leaves **A**, **C***P.piliferum* (*U. Laine*, TUR!) **B**, **D***P.latebricola* (from lectotype of P.latebricolavar.gemmascens, PC 0132685!).

Plants small to medium sized, light green to yellowish green; leaves more or less complanate, ovate to lanceolate, concave, symmetrical, 0.8–1.5 × 0.4–0.8 (Fig. 12A), abruptly narrowed to a long filform acumen; the apex denticulate; laminal cells linear, 40–110 × 5–7 μm at midleaf (Fig. 12C), cell areolation tight; decurrencies of 2–3 rows of cells; setae 0.8–1.5 cm, capsule erect.

###### Distribution.

Asia (China, Democratic People’s Republic of Korea, Japan, Republic of Korea, Russia Federation, Turkey); Europe (Andorra, Denmark, Finland, France, Ireland, Italy, Latvia, Norway, Portugal, Romania, Slovenia, Spain, Sweden, Switzerland, Ukraine, United Kingdom); North America (Canada, U.S.A.).

#### Sect. Philoscia (Berk.) Ochyra

##### ﻿ Plagiothecium latebricola

Taxon classificationPlantaeHypnalesPlagiotheciaceae

Wilson ex Schimp., Bryologia Europea 5: 184, 494, Tab. I. 1851.

1063186B-246B-5E0C-AF16-4245E39BFFC2

≡ Leskealatebricola (Schimp.) Wilson, Bryologia Britannica 329, 54. 1855 ≡ Philoscialatebricola (Schimp.) Berk., Handbook of British Mosses 146. 1863 ≡ Hypnumlatebricola (Schimp.) Lindb., Bidrag till Sydöstra Tavastlands Flora 154. 1870 ≡ Isopterygiumlatebricola (Schimp.) Delogne, Annales de la Société Belge de Microscopie 9: 141. 1885 ≡ Plagiotheciellalatebricola (Schimp.) M.Fleisch. *in* Brotherus, Die natürlichen Pflanzenfamilien, Zweite Auflage, 11: 466. 1925. Type: [Great Britain,] in truncis Alnorum semiputridis prope Hurstpierpoint (Sussex) ubi el. Mitten primus parcissime legit; prope Warrington (Wilson).
= Plagiotheciumlatebricolavar.gemmascens Ryan & I.Hagen, Kongelige Norske Videnskabers Selskabs Skrifter 1896(1): 135. 1896 [1897] ≡ Plagiotheciumlatebricolafo.gemmascens (Ryan & I.Hagen) Correns, Untersuchungen über die Vermehrung der Laubmoose 248. 1899 ≡ Plagiotheciellalatebricolafo.gemmascens (Ryan & I.Hagen) Podp., Conspectus Muscorum Europaeorum 682. 1954. Type: Nordlands ved Åle i Onsø (oktober 1889: R.) også funden ved vejen malle Larvik og Fredriksvaern, på rådne orestubber i en myr, (1/8 1890: kand. E. Nyman) og ved Rognan i Saltdalen, under dryppet fra tagskjaegger på vaeggen af et bådnøst (30/8 1892: H.). Lectotype (designated here): Nordlands amt, Salten, Saltdalen, Rognanm ad lignum vetustum in stillicides, 67°5'N, 30/8 1892, Musci Norvegici ex. herb. *I. Hagen*, PC 0132685!

###### Description.

Plants small, slender, bright green to yellowish-green; leaves complanate, narrowly ovate-lanceolate, symmetrical, 0.7–1.2 × 0.3–0.5 mm (Fig. 12B); the apex long acuminate; margins denticulate near the apex or not, gemmae often present on apex or leaf axils; laminal cells very narrow, 80–130 × 5–7 μm at midleaf (Fig. 12D), cell areolation tight; decurrencies of 2–3 rows of rectangular cells; setae 0.8–1.2 cm, capsule erect.

###### Distribution.

Asia (China, Georgia, Japan, Kyrgystan, Pakistan, Russian Federation, Sri Lanka, Turkey); Europe (Austria, Belarus, Belgium, Czech Republic, Denmark, Estonia, Finland, France, Germany, Hungary, Ireland, Italy, Latvia, Lithuania, Luxembourg, Netherlands, Norway, Poland, Portugal, Romania, Serbia, Slovakia, Sweden, Switzerland, Ukraine, United Kingdom); North America (Canada, U.S.A.).

#### Sect. Pseudo-Neckera (Kindb.) J.T.Wynns

##### ﻿ Plagiothecium neckeroideum

Taxon classificationPlantaeHypnalesPlagiotheciaceae

Schimp., Bryologia Europea 5: 194, 505, Tab. XII. 1851.

AF913030-FBD7-5077-8962-9F5E28AEFE26

≡ Stereodonneckeroideus (Schimp.) Mitt., Journal of the Proceedings of the Linnean Society, Botany, Supplement 1(2): 103. 1859 ≡ Hypnumneckeroideum (Schimp.) Lindb., Animadversiones de Hypno elegante 28. 1867, nom. inval. Type: [Austria], Loco praerupto umbroso ad viam supra catarractum Krimml-Fall dicta Alpinum salisburgensium, ubi. *W. P. Sch.* Julio 1843 detexit.

###### Description.

**Figure 13.**
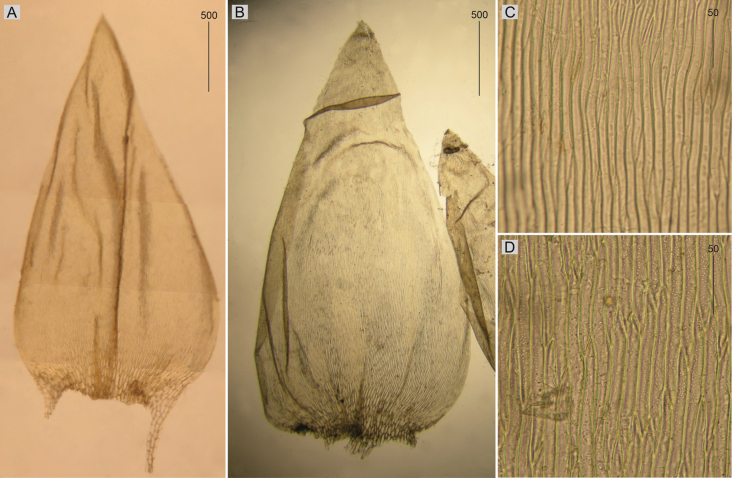
Selected, most important taxonomic features of the *Plagiotheciumneckeroideum* and *Plagiotheciumundulatum***A, B** shape and dimensions of the leaves **C, D** shape and dimensions of cells from the middle part of the leaves **A**, **C***P.neckeroideum* (from lectotype of P.neceroideumvar.mureum, *Holler*, C-M-9389! and syntype of P.neceroideumvar.javense, *M. Fleischer*, PC 0132631!, PC 0132632!) **B**, **D***P.undulatum* (based of *P.menziesii*, *A. Menziesi*, PC 0132669!).

Plants large, light green to yellowish green; stems 2–4 cm long; leaves of two types: ventral and dorsal symmetrical and asymmetrical, lateral ones distinctly asymmetrical, ovate, concave, undulate, 1.5–2.8 × 0.9–1.8 mm (Fig. 13A); apex acute; margins denticulate near the apex; laminal cells linear, 70–100 × 5–7 μm at midleaf (Fig. 13C), cell areolation tight; decurrencies of 3–4 rows of rectangular to quadrate cells; setae 1.5–2.0 cm; capsules inclined or almost erect.

###### Distribution.

Asia (Bhutan, China, Democratic People’s Republic of Korea, India, Indonesia, Japan, Malaysia, Nepal, Philippines, Republic of Korea, Russian Federation, Taiwan, Thailand); Europe (Austria, Czech Republic, Germany, Romania, Slovenia, Switzerland, Ukraine).

#### Sect. Lycambium Jedl.

##### ﻿ Plagiothecium undulatum

Taxon classificationPlantaeHypnalesPlagiotheciaceae

(Hedw.) Schimp., Bryologia Europea 5: 195, 506, Tab. XIII. 1851.

B230E9C9-D785-53C0-B691-53D3B2A52BD2

≡ Hypnumundulatum Hedw., Speciorum Muscorum Frondsorum 242. 1801 ≡ Stereodonundulatus (Hedw.) Mitt., Journal of the Linnean Society, Botany 8: 39. 1865 [1864] ≡ Pancoviaundulata (Hedw.) J.Kickx f., Flore Cryptogamique des Flandres 1: 93, 1867 ≡ Neckeropsisundulata (Hedw.) Kindb. *ex* J.A.Allen, Mosses of the Cascade Mountains, Washington 117. 1900, *hom. illeg.*, *non* (Hedw.) Reichardt ≡ Buckiellaundulata (Hedw.) Ireland, Novon 11(1): 55. 2001. Type: Ad terram humidiusuclam sylvarum umbrosarum planitiei et montium totius Europae. Lectotype (designated by Ireland 1969): In silvis densis acerosis ad terram, in cavernosis saxosis Europae, in Hercynia, Franconia, G 00040241!
= Plagiotheciummenziesii Thér. *ex* J.T.Wynns, in sched. Based on: New Zealand, *A. Menziesi*, *ex hab*. *P. E. Boissier*, cum Hypnummolluscum, *ex herb*. *I.Thériot*, PC 0132669! syn. nov.

###### Description.

Plants large, whitish-green; stems 3–9 cm long, more or less complanate-foliate; leaves transversely undulate, symmetrical to slightly asymmetrical, imbricate, ovate, 2.5–4.5 × 1.3–2.5 mm (Fig. 13B); the apex acute to obtuse, denticulate or not; laminal cells papillose, 90–175 × 7–10 μm at midleaf (Fig. 13D), cell areolation tight; decurrencies of 1–3 rows of rectangular to quadrate cells; setae 2.5–4.5 cm, capsule inclined.

###### Distribution.

Asia (Azerbaijan, China, Islamic Republic of Iran, Russian Federation, Turkey); Europe (Austria, Belarus, Belgium, Bosnia and Herzegovina, Bulgaria, Croatia, Czech Republic, Denmark, Estonia, Finland, France, Hungary, Ireland, Italy, Latvia, Lichtenstein, Lithuania, Luxembourg, Montenegro, Netherlands, Norway, Poland, Portugal, Romania, Serbia, Slovakia, Slovenia, Spain, Sweden, Switzerland, Ukraine, United Kingdom); North America (Canada, U.S.A.).

### ﻿Key to European taxa of *Plagiothecium*

**Table:** 

1	Decurrencies narrow or very narrow, wedge-shaped, composed only of square and rectangular cells, very often remaining attached to stem on dissection	**2**
–	Decurrencies wider, forming distinct or semidistinct auricles, composed of square, rectangular, rounded and inflated cells or only rounded and inflated cells, decurrencies usually attached to the leaf on dissection	**22**
2	The cells of the middle part of the leaves narrow, 10 µm or less, cell areolation tight	**3**
–	The cells of the middle part of the leaves narrow to wide, 10 µm or more, cell areolation tight to loose	**10**
3	Leaves symmetrical or almost symmetrical, but always one type of leaves	**4**
–	Leaves asymmetrical or two types of leaves – symmetrical and asymmetrical	**6**
4	Plants small size, 2–6 cm long	**5**
–	Plants large size, 5–13 cm long	***P.undulatum***
5	Leaves gradually tapering to apex	***P.latebricola***
–	Leaves abruptly narrowed to long, filiform acumen	***P.piliferum***
6	Leaves asymmetrical	**7**
–	There are two types of leaves on the stem, symmetrical and asymmetrical	**9**
7	Plants small size, 1.5–2.0 cm long; leaves lanceolate, concave; apex often denticulate by 2–3 teeth	***P.laetumvar.hercinicum***
–	Plants small or even smaller, 0.6–2.0 cm long; leaves ovate-lanceolate, rather flat; apex rather entire	**8**
8	Leaves forming 40–100° angle with stem, flat, short and narrow, 0.6–1.6 × 0.3–0.6 mm; setae short, about 1 cm, capsules more or less slightly inclined	***P.rossicum***
–	Leaves forming 20–70° angle with stem, concave, longer and wider, 1.0–2.0 × 1.1–1.3 mm; setae longer, 1.3–1.8 cm; capsules erect	***P.laetumvar.laetum***
9	Plants dark green; leaves not undulate and not folded; the apex often gently curved; margins not denticulate near the apex	***P.svalbardense***
–	Plants light green to yellowish green; leaves undulate and folded; the apex straight; margins denticulate near the apex	***P.neckeroideum***
10	Leaves symmetrical	**11**
–	Leaves asymmetrical or gently asymmetrical	**20**
11	Stems erect	***P.berggrenianum***
–	Stems creeping	**12**
12	Leaves flat or slightly concave	**13**
–	Leaves clearly concave	**16**
13	The cells of the middle part of the leaf short and wide, 50–90 × 17–20 μm	***P.nemorale***
–	The cells of the middle part of the leaf long, very long and wide, 130–260 × 10–22 μm	**14**
14	Plant usually yellowish gold, golden green, golden; leaves large, 2.50–3.00 × 0.80–1.40 mm; laminal cells 130–240 × 10–18 μm at mid-leaf	***P.succulentumvar.succulentum***
–	Plant usually dark golden to brown; leaves and laminal cells of other dimensions	**15**
15	Leaves ovate, in dry condition shrunken, not folded, long and wide, 3.0–3.60 × 1.40–1.60 mm; apex acuminate	***P.succulentumvar.propaguliferum***
–	Leaves lanceolate, not shrunken in dry condition, folded, long and narrow, 1.9–3.5 × 0.6–1.0 mm; apex abruptly narrowed to long filiform acumen	***P.succulentumvar.cryptarum***
16	Leaves with an eroded apex	***P.sakuraii***
–	Leaves without an eroded apex	**17**
17	Leaves serrate	***P.ikegamii***
–	Leaves not serrate	**18**
18	Capsules inclined	**19**
–	Capsules erect	***P.flaccidum***
19	The cells from the middle part of the leaf to 101 µm in length	***P.cavifolium***
–	The cells from the middle part of the leaf more than 101 µm in length	***P.subjulaceum***
20	Plants medium-sized to large; leaves large, 3–4 × 1.6–2 mm, distinctly concave, very asymmetrical; cells very wide, 17.0–34.0 μm, cell areolation very loose	***P.longisetum***
–	Plants with a different combination of these features	**21**
21	Plants medium-sized, stems 2–4 cm long; leaves concave, folded, julaceous and imbricate mainly on lower part of the stem, quite large, 3.1–3.4 × 1.3–1.5 mm; the apex acuminate, short; laminal cells quite long and wide, 113–143.3 × 15.1–19.3 μm at midleaf	***P.angusticellum***
–	Plants small, stems 0.5–1.5 cm long; leaves flat, not folded or imbricate and not julaceous, very small, 1.2–1.8 × 0.6–0.8 mm; the apex acuminate, long; laminal cells short and quite narrow, 70–100 × 10–12 μm at midleaf	***P.tenue***
22	Decurrencies quite narrow but not wedge-shaped, forming semidistinct auricles, composed of square, rectangular, rounded and inflated cells, however, square and rectangular cells clearly dominate	**23**
–	Decurrencies forming clear, wide, shorter or longer auricles, composed of rounded and inflated cells	**26**
23	Plants rather medium-sized; leaves complanate, not cracked at the base	**24**
–	Plants medium-sized or small; leaves julaceous and imbricate, mainly in lower part of the stem, often cracked at the base	**25**
24	Leaves symmetrical, long and wide, 1.7–2.7 × 0.7–1.5 mm; apex not hooked, and not curved towards the ground, usually not denticulate; cells from midleaf 110–151 × 8–9 μm	***P.curvifoliumvar.curvifolium***
–	Leaves asymmetrical, long and narrow, 1.7–2.2 × 0.6–0.9 mm; apex hooked, curved towards the ground, usually denticulate by 2–3 teeth; cells from midleaf 60–120 × 7–9 μm	***P.curvifoliumvar.recurvum***
25	Plants medium-sized, leaves julaceous and imbricate mainly in lower part of the stem; leaves asymmetrical; cells from midleaf 95–190 × 6–10 μm	***P.decursivifolium***
–	Plants small, clearly julaceous and imbricate; two types of leaves, symmetrical and asymmetrical; cells from midleaf 80–190 × 5–9 μm	***P.imbricatum***
26	Two types of leaves on the stem, the symmetrical ones: rounded symmetric, with two rounded sides, and asymmetrical ones: with one rounded and one flattened side	***P.denticulatumvar.pseudosylvaticum***
–	Only symmetrical or only asymmetrical leaves on the stem	**27**
27	Only symmetrical leaves on the stem	**28**
–	Only asymmetrical leaves on the stem	**29**
28	Plants medium-sized; leaves imbricate, julaceous, concave; apex not eroded	***P.denticulatumvar.pungens***
–	Plants large; leaves not imbricate and not julaceous, more or less flat; apex often eroded	***P.sylvaticumvar.sylvaticum***
29	Plants medium-size to large, stems 2–5 cm long; leaf apex acute to acuminate, usually denticulate; leaves long and wide, 1.4–3.0 × 0.5–3.6 mm	30
–	Plants small, stems 0.9–2.5 cm long; leaf apex obtuse, not denticulate; leaves short and narrow, 1.0–2.2 × 0.5–1.2 mm	***P.denticulatumvar.obtusifolium***
30	Leaves not shrunken when dry, not transversely undulate, ovate, with two rounded sides	**31**
–	Leaves shrunken when dry, transversely undulate, ovate to ovate-lanceolate, with one rounded and one flattened side	***P.denticulatumvar.undulatum***
31	Leaves more or less complanate-foliate, julaceous in lower part of stem, 1.5–3.0 × 0.5–2.0 mm; the apex not eroded	***P.denticulatumvar.denticulatum***
–	Leaves not overlapping, not imbricate and not julaceous, 3.4–3.6 × 1.4–2.0 mm; the apex often eroded	***P.sylvaticumvar.immersum***

## ﻿Discussion

The ambiguous taxonomic status of individual species of the genus *Plagiothecium* which have been widely described in the literature over the last decades (Nyholm 1965; Lewinsky 1974; Noguchi 1994; Smith 2001) results from several facts. First of all, from the too hasty synonymization of many names, which in later years led to a reduction in the number of distinguished species and to an overly broad treatment of the remaining ones (Ireland 1969, 1985; Iwatsuki 1970).

The perception of *Plagiothecium* by subsequent generations of bryologists was also significantly influenced by which taxonomic features were considered diagnostic. At the same time, each of the commonly recognized studies considered the width of the cells of the middle part of the leaf as one of the first and most important taxonomic features distinguishing individual species (e.g., Greene 1957; Nyholm 1965; Smith 2001). Thus, in the narrow-cell group there were, e.g., *P.laetum* and *P.curvifolium* and in the wide-cell group, among others, *P.nemorale* and *P.denticulatum*. However, the latter two (*P.nemorale* and *P.denticulatum*) are sometimes difficult to distinguish in poorly prepared leaves, without preserved and analyzed decurrencies, and consequently errors of determination of individual taxa are quite frequent (Wolski and Nowicka-Krawczyk 2020).

The above-mentioned decurrencies and their significant role in the discrimination of individual species, including the division of the genus into sections, were already indicated by Jedlička (1948, 1950), although subsequent keys and revisions did not attach such great importance to this feature.

An equally important issue, very rarely mentioned, which Wolski et al. (2022a) noticed recently, is the possibility of two types of leaves existing on one plant — symmetrical and asymmetrical. This, together with other qualitative and quantitative features, allowed the description of a new species – *Plagiotheciumimbricatum* (Wolski et al. 2022a) and, in this study, to propose a new taxon within the *P.denticulatum* complex: P.denticulatumvar.pseudosylvaticum.

The new combinations proposed here are justified because not only are they easily distinguished from other closely related taxa, but also their presence and subsequent separation within individual complexes explains the outstanding variability of these taxa described in the literature (Lewinsky 1974; Noguchi 1994; Smith 2001; Cano 2018). Thus, P.denticulatumvar.pseudosylvaticum and P.denticulatumvar.pungens differ from other members of the *P.denticulatum* complex, e.g., by shape, concavity, symmetry of leaf and dimensions of the cells from the middle part of the leaf. Plagiotheciumlaetumvar.hercinicum is distinguished within the *P.laetum* complex, e.g., by plant size, shape, size, concavity of leaf, apex serration, and dimensions of the cells from the middle part of the leaf. Plagiotheciumsucculentumvar.cryptarum differs from other taxa within the *P.succulentum* complex, e.g., by the color of the plant, the shape, dimensions, leaf folding, and the shape and length of the apex. On the other hand, P.sylvaticumvar.immersum differs from P.sylvaticumvar.sylvaticum in the color of the turf, the symmetry and dimensions of the leaves, as well as the dimensions of the cells from the middle part of the leaf. Due to these features, as well as the descriptions given above, these taxa can be quite easily distinguished macroscopically and microscopically from other closely related species.

*Plagiotheciumruthei* is a taxon morphologically and genetically distinct from other representatives of the *P.denticulatum* complex (Wynns 2015; Wynns et al. 2017). This name (*P.ruthei*) is widely recognized by many bryologists and easily associated with features associated with this species. But, contrary to the cited literature (Wynns 2015; Wynns et al. 2017), I propose, as suggested by Hill et al. (2006) and Blockeel et al. (2020), to treat it as a variety of *P.denticulatum* — P.denticulatumvar.undulatum. This is related to the availability of the oldest name referring to this taxon. A similar situation has been documented, e.g., by Iwatsuki (1970) in the context of *P.cavifolium* (= *P.roeseanum*) or by Wolski et al. (2024) in the context of *P.sylvaticum* (= *P.platyphyllum*).

In the current list, given from Europe by Wynns and Schröck (2018), *Plagiotheciumhandelii* Broth. was not included as a member of the European flora, because the material presented by these authors deviates from the type specimens of this species (isolectotype CP0132634!, syntype CP0132633!) and is more similar to *P.angusticellum* which was described in 2020 (Wolski and Nowicka-Krawczyk 2020).

In this article ten lectotypes are designated for: P.denticulatumvar.bullulae, P.denticulatumvar.hercynicum, P.latebricolavar.gemmascens, P.platyphyllumfo.immersum, P.succulentumfo.propaguliferum, P.succulentumvar.longifoliumfo.splendens, P.sylvaticumfo.pungens, P.sylvaticumvar.cryptarum, P.sylvaticumvar.flavescens and P.sylvaticumvar.rupestre, formally ending the taxonomic revision of these names (Wolski and Proćków 2021).

## Supplementary Material

XML Treatment for Plagiothecium denticulatum var. denticulatum

XML Treatment for Plagiothecium denticulatum var. obtusifolium

XML Treatment for Plagiothecium denticulatum var. undulatum

XML Treatment for Plagiothecium denticulatum var. pseudosylvaticum

XML Treatment for Plagiothecium denticulatum var. pungens

XML Treatment for Plagiothecium sylvaticum var. sylvaticum

XML Treatment for Plagiothecium sylvaticum var. immersum

XML Treatment for Plagiothecium nemorale

XML Treatment for Plagiothecium longisetum

XML Treatment for Plagiothecium angusticellum

XML Treatment for Plagiothecium succulentum var. succulentum

XML Treatment for Plagiothecium succulentum var. propaguliferum

XML Treatment for Plagiothecium succulentum var. cryptarum

XML Treatment for Plagiothecium cavifolium

XML Treatment for Plagiothecium ikegamii

XML Treatment for Plagiothecium subjulaceum

XML Treatment for Plagiothecium flaccidum

XML Treatment for Plagiothecium tenue

XML Treatment for Plagiothecium berggrenianum

XML Treatment for Plagiothecium svalbardense

XML Treatment for Plagiothecium curvifolium var. curvifolium

XML Treatment for Plagiothecium curvifolium var. recurvum

XML Treatment for Plagiothecium decursivifolium

XML Treatment for Plagiothecium imbricatum

XML Treatment for Plagiothecium laetum var. laetum

XML Treatment for Plagiothecium laetum var. hercinicum

XML Treatment for Plagiothecium rossicum

XML Treatment for Plagiothecium piliferum

XML Treatment for Plagiothecium latebricola

XML Treatment for Plagiothecium neckeroideum

XML Treatment for Plagiothecium undulatum

## ﻿Additional information

### Author ORCIDs

Grzegorz J. Wolski https://orcid.org/0000-0003-1480-8003

### Data availability

All of the data that support the findings of this study are available in the main text.
